# Proteogenomics Reveal the Overexpression of HLA-I in Cancer

**DOI:** 10.1021/acs.jproteome.3c00491

**Published:** 2023-10-19

**Authors:** Ying Wang, David Fenyö

**Affiliations:** † Institute for Systems Genetics, NYU Grossman School of Medicine, New York, New York 10016, United States; ‡ Department of Biochemistry and Molecular Pharmacology, NYU Grossman School of Medicine, New York, New York 10016, United States

**Keywords:** HLA-I, CPTAC, mass spectrometry, cancer, protein expression

## Abstract

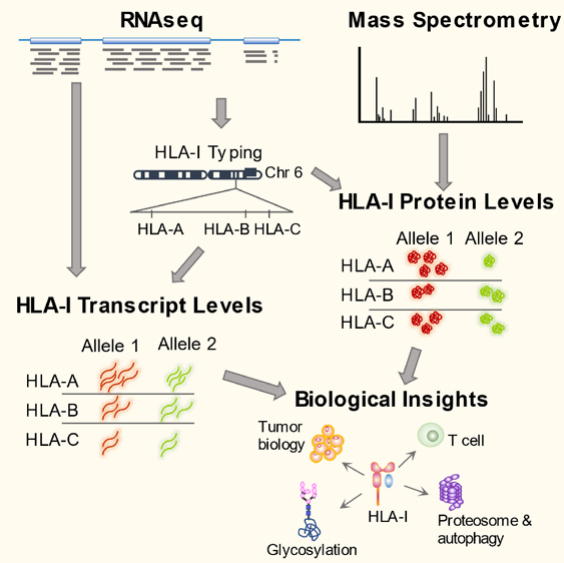

An accurate quantification of HLA class I gene expression is important in understanding the interplay with the tumor microenvironment of antitumor cytotoxic T cell activities. Because HLA-I sequences are highly variable, standard RNAseq and mass spectrometry-based quantification workflows using common genome and protein sequence references do not provide HLA-I allele specific quantifications. Here, we used personalized HLA-I nucleotide and protein reference sequences based on the subjects’ HLA-I genotypes and surveyed tumor and adjacent normal samples from patients across nine cancer types. Mass spectrometry using data dependent acquisition data was validated to be sufficient to estimate HLA-A protein expression at the allele level. We found that HLA-I proteins were present in significantly higher levels in tumors compared to adjacent normal tissues from 41 to 63% of head and neck squamous cell carcinoma, uterine corpus endometrial carcinoma, and clear cell renal cell carcinoma patients, and this was driven by increased levels of HLA-I gene transcripts. Most immune cell types are universally enriched in HLA-I high tumors, while endothelial and neuronal cells showed divergent relationships with HLA-I. Pathway analysis revealed that tumor senescence and autophagy activity influence the level of HLA-I proteins in glioblastoma. Genes correlated to HLA-I protein expression are mostly the ones directly involved in HLA-I function in immune response and cell death, while glycosylation genes are exclusively co-expressed with HLA-I at the protein level.

## Introduction

During malignant transformation and cancer progression, mutations accumulated in the cells are potential sources of immunogenic neo-antigens. These antigens are presented by MHC class I molecules and are recognized by cytotoxic T cells, which consequently destroy tumor cells. However, tumors develop strategies to escape such immune surveillance. HLA-I loss is believed to be one of the major mechanisms, and it was reported in multiple cancer types and identified as the cause of acquired resistance to immune checkpoint inhibitor therapy.^1−3^ Several aspects of HLA-I loss have been described, including the loss of heterozygosity (LOH), HLA gene methylation, mutations, and transcriptional regulation on HLA genes and β2-microglobulin (B2M).^4^

Next-generation sequencing (NGS) has enabled HLA typing and detection of HLA LOH events across many tumor samples. Numerous algorithms have been developed to determine genotypes of HLA-I and identify LOH events with widely available whole-genome, exome, and RNA sequencing data.^5^ Seventeen percent of tumors were estimated to harbor somatic HLA LOH across different cancer types.^6^ How a lost allele on the genomic level impacts the HLA-I transcript and protein expression profile has not been well described though. Often B2M gene expression either at the RNA level or protein level is used as the sole representation of the HLA molecules, ignoring the fact that B2M expression may not reflect the level of HLA-I heterodimers; especially, antigen binding does not involve direct contact with B2M but only on HLA-A, HLA-B, and HLA-C molecules. RNAseq has been widely used to quantify gene expression in tumors, but HLA-I sequences are inadequately represented in genome references used in standard pipelines. HLA-I gene sequences are highly diverse with 6000–7500 genomic sequences and 3000–4500 different protein sequences.^7^ HLA-I protein expression is mostly estimated by immunohistochemistry assays using anti-HLA-I antibodies lacking differentiation of different genotypes. Currently, mass spectrometry (MS)-based proteomic methods are the only options for high-throughput proteome quantification. However, MS studies targeted to HLA-I molecules are rare, and the landscape of the HLA-I protein in cancer remains obscure. CPTAC studies profiled numerous tumor and adjacent normal samples with both RNAseq and MS proteomic assays. This provided us an opportunity to investigate the expression level of HLA-I on both the RNA and protein levels from the same samples. We determined the genotype of patient HLA-I using RNAseq data and then used the typing information to evaluate the feasibility of quantifying allele-specific HLA-I expression with MS data. We identified the aberrant expression pattern in tumors by comparing with adjacent normal samples and investigated the consistency of such patterns on the RNA and protein level. Furthermore, we explored the association of tumor HLA expression with various tumor features, including the cell-type composition of the tumor and biological pathways.

## Experimental Procedures

### Data Collection

#### RNAseq

The harmonized whole transcriptome mRNA sequencing data generated by the Clinical Proteomic Tumor Analysis Consortium (CPTAC) were downloaded from the Genomic Data Commons' Data Portal (https://portal.gdc.cancer.gov/). Genomic BAM files from the two-pass method with STAR were used to recover all original reads. Altogether, RNAseq data from 1328 tumor and adjacent normal samples from 979 patients in CPTAC2 and CPTAC3 studies of nine cancer types were used in this study.

#### Mass Spectrometry-Based Proteomics

The raw mass spectrometry files from CPTAC2 and CPTAC3 studies were downloaded from the CPTAC Data Portal (https://cptac-data-portal.georgetown.edu/cptac/public) and converted to the mzML format using the MSConvert tool from the ProteoWizard project (proteowizard.sourceforge.net). All mass spectrometry files used in this study were acquired using data dependent acquisition (DDA) with TMT10plex or TMT10 plus TMT11–131C isobaric labels. Altogether, 4099 whole proteome spectrum files from 1461 tumor and adjacent normal samples from 963 patients in CPTAC2 and CPTAC3 studies of nine cancer types were used in this study.

### Identification and Quantification of Proteomic Data

For each of the nine CPTAC2 and CPTAC2 studies, global proteomic mass spectrometry data from 11 to 24 TMT10 or TMT11 multiplex assays, each with 12–25 RPLC fractions, were analyzed independently. X!Tandem release 2017.10.03^8^ was used to match identified peptides in mzML spectrum files against the Ensembl release100 human protein sequence database^9^ downloaded on August 10, 2020 (hg38, 111060 entries), combined with all HLA sequences from IMGT/HLA, that is, 116 common containments from laboratory reagents. All spectra were allowed a ±20 ppm monoisotopic mass tolerance for parent and fragment ions. Allowed modifications included carbamidomethylation (+57.0215 Da) of cysteine and selenocysteine, TMT labeling (+229.1629 Da) at lysine and peptide N-termini, and dynamic oxidation (+15.9949 Da) of methionine. Refinement modifications include acetylation of peptide N-termini, oxidation of methionine and tryptophan, and deamidation of asparagine and glutamine.

An “expectation” value is estimated by scoring a peptide match with the distribution cyclic permutation and search on reversed reference sequences.^10^ A threshold of an expectation value of 0.001 to identify a high-confidence peptide match was used. All identified peptides with high confidence for each study are listed in Table S1, and all unique proteins identified for proteome for each study are listed in Table S2.

The intensities of TMT reporter ions were obtained by converting mzML files to the mgf format using the MSConvert tool from ProteoWizard, and then we extracted the intensity of mass matching the TMT reporter mass provided by the TMTplex vendor ThermoFisher. Peptides identified with at least one reporter were selected. Reporter ion intensities for each identified peptide including different charges were grouped together from different scans and different RPLC fractions. A common pooled reference sample was labeled with one of the TMT10 or TMT11 reporters, and the reporter intensities from this reporter were used to compare the peptide abundance across all samples in the same assay. Low-quality samples reported in each of the studies^11−16^ were removed from all analysis. For whole-proteome quantification, the reporter ion intensities from all peptides uniquely mapped to a gene were summed and the relative protein abundance was calculated as the ratio of such summed intensities of a sample to the common pooled reference sample. This relative abundance ratios were then log2-transformed and median-centered across all samples in the same study.

### Experimental Design and Statistical Rationale

#### HLA Typing

Genotypes of HLA class I genes HLA-A, HLA-B and HLA-C were determined with arcasHLA software.^17^ Briefly, reference coding DNA sequences of HLA class I genes were constructed with sequences obtained from the ImMunoGeneTics/HLA database, IMGT/HLA.^7^ Only full-length HLA sequences were used for genotyping. To avoid missing HLA gene reads not aligned properly to the standard genome reference, all unmapped reads were extracted and included for HLA genotyping together with the ones mapped to Chromosome 6 as input. Pseudoalignments of extracted reads to reference HLA sequences were performed with Kallisto, a de Bruijn Graph (T-DBG) based transcript-level RNAseq quantification tool.^18^ An iterative procedure ranked and selected the most likely genotypes by most reads explained until convergence. Homozygous alleles were called with a threshold of 15% of a minor-to-major ratio of non-shared allele counts. Allele typing was done at three-field resolution (coding sequence level) and then converted to two-field (protein level) nomenclature in the World Marrow Donor Association (WMDA) format. To verify the quality of the HLA typing result, HLA genotypes from paired tumor and adjacent normal samples in four studies were compared to identify discrepancies. Out of 333 tumor normal pairs, 17 pairs had a difference in genotypes and all but 1 such discrepancy were lost of heterozygosity in tumors.

#### RNA Expression

All non-redundant paired-end RNAseq reads were extracted from alignment bam files and saved in the fastq format as input. Gencode v 35^19^ (https://www.gencodegenes.org/human/) was used as a common transcript reference. To construct an individual transcript reference for each sample with its specific HLA gene sequences, all sequences of HLA class-I genes were removed from the common transcript reference and replaced with the coding sequences of the specific HLA alleles of the sample. Using the HLA allele supplemented transcript reference, the RNA level expression was quantified with Kallisto^18^ using default settings with a sequence-based bias correction. Gene-level RNA counts were estimated by taking the sum of all transcripts of the same gene. Variance stabilizing transformation (VST) from R package DESeq2^20^ was then applied to the count data and normalized with respect to the library size for downstream analysis. Data from each of the nine studies were processed and normalized independently. HLA class I genes were quantified separately for each allele, and the total expression of the gene was determined by the log sum of the two alleles if the sample is heterozygous.

#### HLA Class I Protein Expression

Peptides matching sequences from non-HLA genes or multiple HLA genes were eliminated from the analysis. All identified HLA-I gene peptides with confidence are listed in Table S3. For each sample, the HLA-A, HLA-B, and HLA-C genotype were converted to protein-level nomenclature, and peptides matching the corresponding protein sequences of the two alleles of the sample were used to estimate HLA protein expression. The log2-transformed ratio of the sample to pooled reference was calculated with the sum of corresponding reporter intensities from the allele matching peptides.

#### Molecular Characterization of Tumors

All molecular characterizations of tumors were done using RNAseq expression data summarized at the gene level and transformed with the DESeq2 R package. Data from each of the CPTAC studies are analyzed separately. xCell cell-type enrichment analysis^21^ was performed using all default parameters. SSGSEA analysis was conducted using the GSVA R package^22^ with the C8 gene signature database from MSigDB.^23^

## Results

### HLA-I Is Homozygous at the RNA Level in Less than 30% of Tumors

The loss of heterozygosity of HLA (HLA LOH) is believed to be one of the mechanism tumors evading immune surveillance, and a transition of tumor cells from HLA-I positive to HLA-I negative was described previously.^1^ We investigate the HLA LOH profile at the mRNA level by genotyping tumor and adjacent normal samples with RNAseq data from nine Clinical Proteomics Tumor Analysis Consortium (CPTAC) studies (Table 1).

**Table 1:** Tumor and Adjacent Normal Counts in Nine CPTAC-2 and CPTAC-3 Studies^a^

		RNAseq		proteomics	
study	cancer	total subjects	paired	total subjects	paired
BRCA	breast cancer	133	0	126	18
CCRCC	clear cell renal cell carcinoma	110	75	117	84
CRC	colorectal cancer	106	0	101	96
GBM	glioblastoma	99	0	100	0
HNSCC	head and neck squamous cell carcinoma	110	61	110	68
LSCC	lung squamous cell carcinoma	108	95	110	102
LUAD	lung adenocarcinoma	111	102	107	97
OV	ovarian high-grade serous carcinomas	101	0	93	10
UCEC	uterine corpus endometrial carcinoma	101	15	104	30

a Samples with quality issues determined by each of the original studies were not included in the analysis and not counted. Total subjects: total number of patients in the study. Paired: the number of patients in the study that have both tumor and adjacent normal samples analyzed.

Out of the paired samples of tumor and adjacent normal from lung adenocarcinoma (LUAD), lung squamous cell carcinoma (LSCC), head and neck squamous cell carcinoma (HNSCC), and clear cell renal cell carcinoma (CCRCC) studies, 5(4.9%), 4(4.2%), 5(8.2%), and 2(2.7%) patients, respectively, presented heterozygous HLA-A, HLA-B, or HLA-C genotypes in the adjacent normal samples while losing one allele in the corresponding tumor samples (Table 2).

**Table 2:** Loss of HLA Heterozygosity in LUAD, LSCC, HNSCC, and CCRCC Tumors^a^

*r*	gene	patient ID	tumor	normal
LUAD	HLA-A	C3L-00279	A*24:02P, A*24:02P	A*25:01P, A*24:02P
	HLA-A	C3N-00169	A*24:03P, A*24:03P	A*24:03P, A*11:01P
	HLA-A	C3N-00580	A*24:07P, A*24:07P	A*03:01P, A*24:07P
	HLA-C	C3N-00580	C*04:01P, C*04:01P	C*04:01P, C*03:04P
	HLA-A	C3N-02155	A*24:02P, A*24:02P	A*01:01P, A*24:02P
	HLA-C	C3N-02155	C*07:02P, C*07:02P	C*07:02P, C*06:02P
	HLA-A	C3N-02586	A*24:02P, A*24:02P	A*24:02P, A*02:06P
LSCC	HLA-A	C3L-01884	A*24:02P, A*24:02P	A*24:02P, A*03:01P
	HLA-A	C3L-02648	A*02:01P, A*02:01P	A*02:01P, A*11:01P
	HLA-A	C3L-03272	A*02:01P, A*02:01P	A*01:01P, A*02:01P
	HLA-A	C3N-01893	A*26:01P, A*69:01P	A*69:01P, A*69:01P
	HLA-A	C3N-02425	A*02:01P, A*02:01P	A*11:01P, A*02:01P
HNSCC	HLA-A	C3L-02651	A*23:01P, A*23:01P	A*23:01P, A*11:01P
	HLA-B	C3N-02275	B*13:02P, B*13:02P	B*13:02P, B*15:01P
	HLA-A	C3N-02279	A*68:01P, A*68:01P	A*68:01P, A*02:01P
	HLA-A	C3N-03841	A*02:01P, A*02:01P	A*11:02P, A*02:01P
	HLA-A	C3N-03928	A*33:01P, A*33:01P	A*33:01P, A*03:01P
CCRCC	HLA-A	C3N-00242	A*24:02P, A*24:02P	A*24:02P, A*03:01P
	HLA-A	C3N-01175	A*23:01P, A*23:01P	A*23:01P, A*03:01P
	HLA-B	C3N-01175	B*08:01P, B*08:01P	B*08:01P, B*35:01P
	HLA-C	C3N-01175	C*07:01P, C*07:01P	C*04:01P, C*07:01P

a The loss of HLA heterozygosity was determined by RNA-level HLA-I genotyping and was defined as an HLA-I gene being heterozygous in the tumor sample and homozygous in paired adjacent normal samples in the same patient.

**Figure 1:**
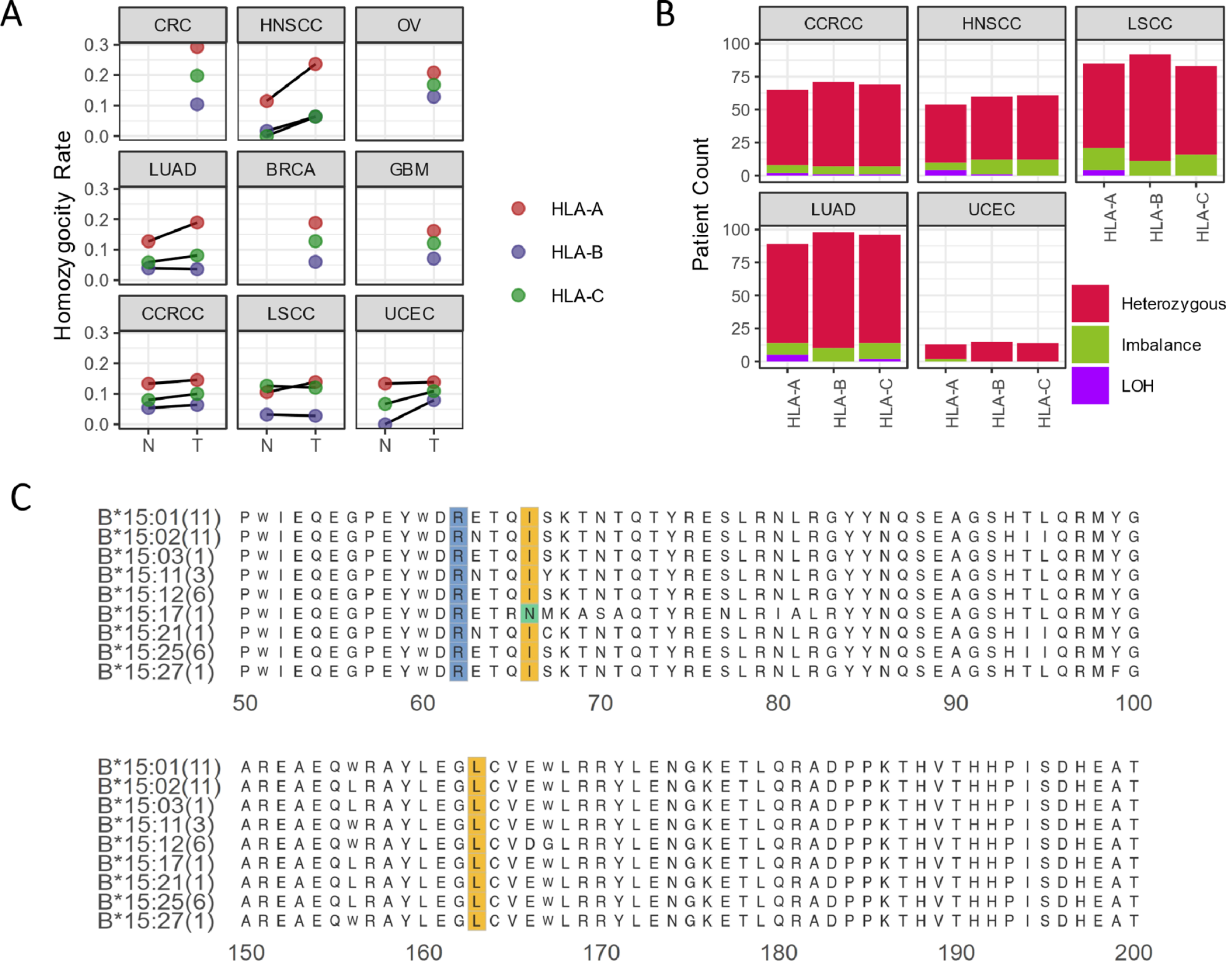
HLA-I are mostly heterozygous in tumors. (A) HLA-I RNA homozygosity rate in tumor and normal samples from nine cancer types. Homozygosity was determined by HLA-I genotyping using RNAseq data with a threshold of 15% for minor alleles. N: adjacent normal; T: tumor. (B) Prevalence of LOH and expression imbalance between two alleles in heterozygous patients from five cancer types. The LOH was determined by HLA-I genotypes comparing tumor and normal samples from the same patient. “Imbalance” is the patients genotyped as heterozygous in both tumor and normal samples but with an RNA expression of one allele less than 50% of the other allele in tumor, and the difference between alleles in tumor was 1.5-fold or higher than the difference between alleles in the corresponding normal samples. (C) Sequence alignment of all B15 alleles in LUAD patients. Highlighted are Arg62, Ile66, and Leu163 forming bridges in the peptide-binding groove. Numbers in parenthesis: number of alleles in LUAD patients.

Only one tumor sample of CCRCC lost one allele in all HLA-A, HLA-B, and HLA-C genes, and two tumor samples of LUAD lost one allele in HLA-A and HLA-C genes. Across all nine cancer types, the homozygosity rate of HLA-I genes in tumor samples ranged from 5 to 30%, and the rate is in general higher than the adjacent normal samples (0–15%) (Figure 1A). Within the same cancer type, the homozygosity rate is consistently the highest for the HLA-A gene (mean = 18.9% across all nine cancers) and the lowest for the HLA-B gene (mean = 7.0%) with the biggest difference around 15% in LUAD and HNSCC tumors. This is concordant with the observation that HLA-A genotypes are the most clonal and the HLA-B gene the most diverse among the general population. The homozygosity rate of HLA-I genes in CPTAC tumors due to the HLA loss at the RNA level is much lower than we expected from previous reports on these cancers. Since bulk RNAseq data lacked the resolution to inspect the HLA homozygosity at the individual cell level, we considered the possibility of intratumor heterogeneity of the HLA-I status contributing to this discrepancy. To this end, we quantified HLA-I gene RNA expression levels for both alleles separately for each tumor sample. When the RNA level of one allele is significantly lower than the other, it suggests an HLA-I loss event in a considerate portion of tumor cells. To accurately quantify each of the two alleles, we constructed a sample-specific, modified reference by removing entries of HLA-I genes from the standard Gencode gene annotation and sequences then supplemented with the sequences of the sample HLA-I genotypes. For all paired tumor and normal samples that harbor heterozygous alleles, we compared the expression-level fold change between two alleles in the same sample (Figure S1). Out of 330 samples across LUAD, LSCC, HNSCC, and CCRCC, only 16 samples showed a fourfold difference in RNA expression between two alleles, while the corresponding normal sample had no more than a twofold difference. We then used a more relaxed criteria to define the “imbalanced” HLA-I expression, that is, the expression of one allele is less than 50% of the other allele in the tumor but not in the adjacent normal. The HLA-I LOH and “imbalanced” altogether account for 0–25% with a mean of 17.3% for HLA-A, 13.0% for HLA-B, and 15.9% for HLA-C, of heterozygous patients (Figure 1B). Therefore, we conclude that intratumor heterogeneity is not likely to be a major contributing factor to the observed discrepancy. These genotyping and RNA expression results suggested that HLA-I LOH is not necessarily a universal feature of tumors across different cancer types at least at the RNA level.

### HLA-B15 Is Overrepresented in Lung Adenocarcinoma Patients

The sequence variations in different HLA-I molecules not only determine their binding specificity for antigen peptides but also affect the binding strength, peptide diversity, HLA-peptide complex stability, and surface expression, resulting in a diverse robustness of immune surveillance against tumorigenesis.^24−26^ We asked the question of whether any HLA allele groups, which are the classification of HLA-I alleles with similar protein sequences, are over- or underrepresented in cancer patients. We calculated the allele frequencies of HLA allele groups in CPTAC patients and compared them with the expected allele frequencies from the general population, adjusting for the race and ethnicity of the patients. Overall, allele frequencies of HLA-A and HLA-C are comparable to the expected values in most of the studies. A few HLA-I allele groups showed higher allele frequencies than expected (multitesting adjusted *p* value <0.05 and over 50% higher than expected allele frequency) including B*13 in GBM, B*18 and C*12 in HNSCC, C*01 in LSCC, and B*15 in LUAD. A*01 in GBM (allele frequency of 5.56%) was lower than expected (12.56%). Interestingly, B*15 is the most prevalent allele group in LUAD patients with an allele frequency of 18.9%, which is 76% more than the expected value (Figure S2). This overrepresentation was not observed in any of the other eight cancer types in which the allele frequency ranges from 4.1 to 13.9%. Previous study of melanoma patients undergoing immune checkpoint blockade (ICB) therapy showed the association of B*15:01 with poor survival.^27^ Molecular dynamics simulations demonstrated that three amino acids (Arg62, Ile66, and Leu163) form a relatively rigid bridge in the peptide-binding groove, which may result in weakened binding to T cell receptors and hence inferior antigen presenting ability. In LUAD patients that harbored a B*15 allele group, all but one patient shared the same three amino acids with B*15:01 (Figure 1C). The overrepresentation of this allele group may suggest a genetic disadvantage of a lower efficiency in presenting neo-antigens in tumorigenic cells to T cells, contributing to the tendency of cancer occurrence. Further investigations are necessary to test this hypothesis.

### Mass Spectrometry Provides Allele-Specific Quantitation of HLA-I Proteins

To investigate whether the existing data-dependent acquisition MS (DDA-MS) data in CPTAC studies is adequate to estimate the allele level abundance of HLA-I proteins, we constructed a sequence database for peptide search including all full-length HLA-I protein sequences from in IMGT/HLA in addition to Ensembl (v100) protein sequences and common contaminants and amino acid modifications. Out of 4822 MS files across nine cancer types, we identified 183–614 unique and HLA-I gene specific peptides for each cancer data set using a stringent cutoff (*p* value <0.001) established using decoy protein sequences (Figure S3a).

All proteomic analyses from the nine CPTAC studies were performed with multiplexed samples using tandem mass tag (TMT)-based isobaric labeling, which increased the complexity in quantifying HLA-I due to shared peptides coming from different samples. To evaluate the sample specificity of the identified peptides, we counted the peptides that match the HLA-I protein sequences derived from the sample HLA-I typing results. For the HLA-A gene, about 100 sample matching peptides were identified for most cancer types, which is 3–5 times the number of HLA-A genotypes (Table 3). At least one genotype-matching HLA-A peptide was identified in over 94% of the patient samples except the ovarian cancer study. This provided sufficient coverage to quantify HLA-A for most of the samples. However, the number of genotype-matching peptides identified for HLA-B and HLA-C was considerably lower, which compromised the quality of quantification for those genes. This is not a surprise since a successful capture of HLA-I peptides requires lysine and arginine at the right interval to produce the right-sized peptides. Different genotypes of HLA-I would have different detectable peptide profiles. HLA genes are also highly homologous; therefore, only peptides derived from the variable region of HLA-I can be used for differentiating different HLA-I genes and alleles. Additionally, the DDA-MS approach used in the CPTAC involves survey scans of precursor peptide ions (MS1) followed by fragmentation of a limited number of selected ions (MS2). Therefore, only a fraction of all precursor peptides in a sample are captured in MS2 for identification and quantification. This further lowers the chance of low-quantity peptides being identified, such as genotype-specific ones not shared with other multiplexed samples in the same assay.

**Table 3:** Number of Unique HLA-I Peptides Detected in Tandem MS Assays from the CPTAC Studies^a^

gene	# allele	# peptide	# patient	% patient
LUAD				
HLA-A	28	132	107	100.0%
HLA-B	52	43	106	99.1%
HLA-C	26	46	107	100.0%
LSCC				
HLA-A	28	136	108	98.2%
HLA-B	53	56	108	98.2%
HLA-C	27	42	108	98.2%
HNSCC				
HLA-A	23	108	108	99.1%
HLA-B	40	45	108	99.1%
HLA-C	26	35	108	99.1%
BRCA				
HLA-A	39	100	121	96.0%
HLA-B	58	50	121	96.0%
HLA-C	27	33	121	96.0%
CCRCC				
HLA-A	27	87	110	94.0%
HLA-B	41	35	107	91.5%
HLA-C	19	15	110	94.0%
CRC				
HLA-A	31	62	96	95.0%
HLA-B	53	16	91	90.1%
HLA-C	25	6	90	89.1%
GBM				
HLA-A	23	104	99	100.0%
HLA-B	44	26	92	92.9%
HLA-C	24	31	97	98.0%
OV				
HLA-A	29	91	82	88.2%
HLA-B	43	23	65	69.9%
HLA-C	28	13	72	77.4%
UCEC				
HLA-A	23	97	100	99.0%
HLA-B	37	34	99	98.0%
HLA-C	19	31	100	99.0%

a HLA-I peptides were identified as described in experimental procedures, and only the peptides to no non-HLA-I gene sequences were counted. # Allele: number of different HLA-I types at protein level in the study. # Peptide: number of unique HLA gene-specific peptides detected. # Patient: number of patients that has HLA gene-specific peptides detected. % Patient: percentage of patients that have HLA gene-specific peptides detected in the study.

**Figure 2:**
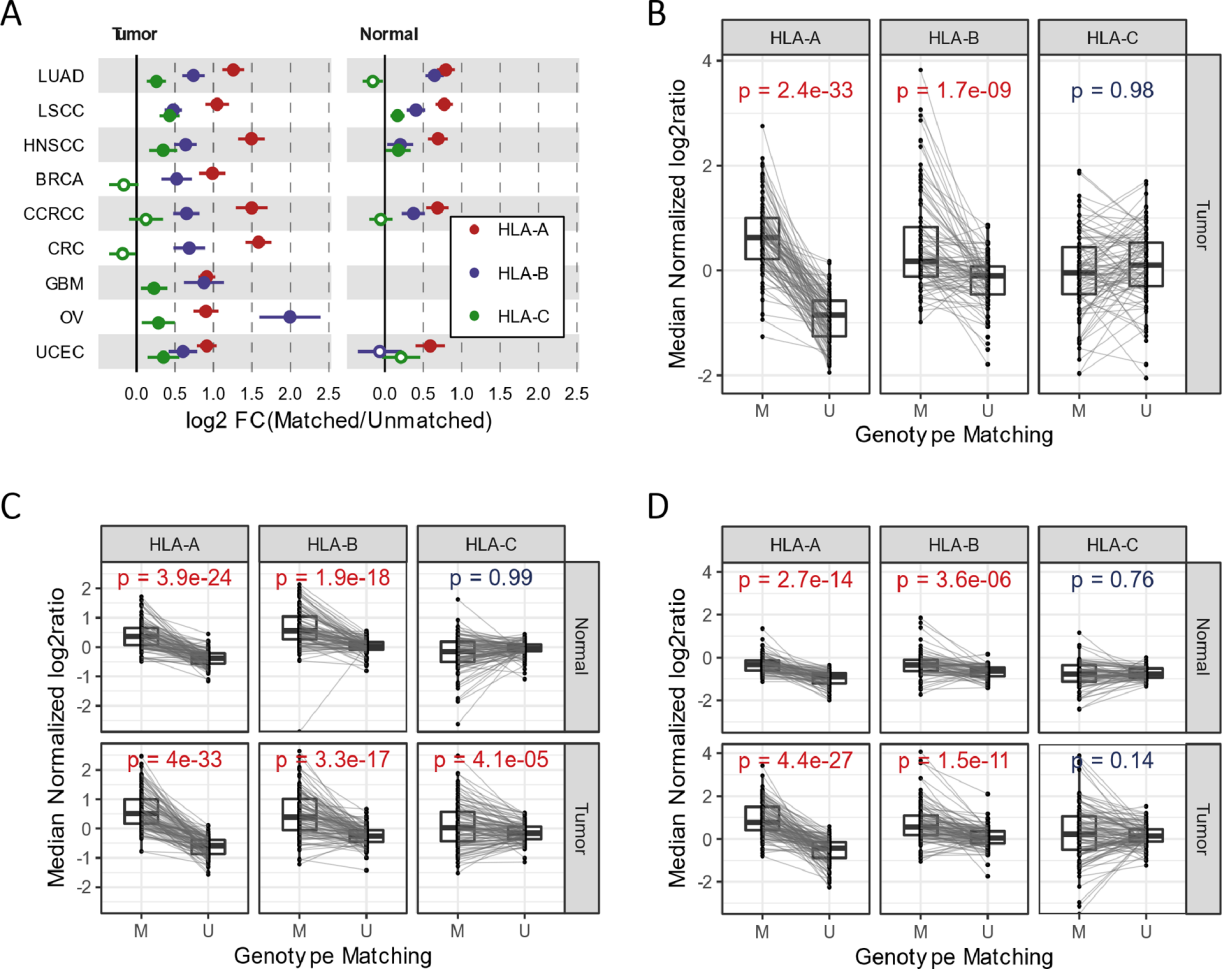
Tandem mass spectrometry provides quantitative protein evidence at the allele level of the HLA-I gene. (A) Expression difference between sample genotype-matched peptides and unmatched peptides. For each of the HLA-I gene specific peptides detected in a sample, the peptide was marked as “matched” if the sequence of the peptide matches the protein sequences of the sample HLA-I type at 100%; otherwise, the peptide was marked as “unmatched”. The expression was quantified by the median normalized log2 ratio of the peptide signal intensity in the sample over the signal intensity from the common reference sample in the study, mixture of multiple tumor and normal samples. The median of the log2 ratio from above for all peptides, either “matched” or “unmatched” in a sample, was used to represent the relative expression of the “matched” and “unmatched” peptides. Fold changes and standard errors (shown as the length the bar for each point on the plot) were established by performing a paired *t* test to compare the relative expression for each HLA-I gene and each study. Solid circle: *p* value <0.05 of the paired *t* test; open circle: not significant. (B–D) Median expression of genotype matched and unmatched peptides in (B) CRC, (C) LUAD, and (D) CCRCC. M: matched peptides. U: unmatched peptides. Each dot represents one sample. *P* values from the paired *t* test comparing the median of all genotype-matched peptides in a sample vs the median of all unmatched peptides of the same sample. Lines connect the expression of matched and unmatched peptides from the same sample.

To evaluate the feasibility of using existing CPTAC proteomic data for the purpose of HLA-I protein expression estimation, we quantified each of the HLA-I peptides using the sample median normalized log-transformed ratio to the common reference sample and compared such ratios between the genotype-matching peptides and non-matching peptides. In all nine cancer types, relative quantities of genotype-matching peptides are significantly higher than non-matching ones for HLA-A (Figure 2 and Figure S3b). The log-transformed fold changes (genotype matching/non-matching) range from 1.87 to 3.00 for tumor samples. The fold changes in normal samples are lower, ranging from 1.51 to 1.73. Fold changes are also significant (paired *t* test) for HLA-B except in UCEC. We also noticed a higher dynamic range of relative quantification of HLA peptides in tumors compared to the adjacent normal, which is concordant with the general observation of diverse protein expression levels due to dysregulation in cancer. Overall, we are confident with using genotype-matching peptide data to estimate HLA-A and HLA-B protein levels, while discretion must be applied for HLA-C. We also counted the number of unique peptides that differentiate two alleles of a sample, that is, the ones matching protein sequences of one allele but not the other. For most of the samples, there are more allele-specific peptides identified for the HLA-A gene with a mean range from 9 to 17 peptides per sample than shared peptides between two alleles (average 5–7 peptides per sample) (Figure S4). This result justified the use of the data set to estimate the allele level expression of this gene. The number of allele-specific peptides is lower for HLA-B and HLA-C with an average of less than five peptides per sample in all studies.

### HLA-I Transcript and Protein levels Are Elevated in Tumors

**Figure 3:**
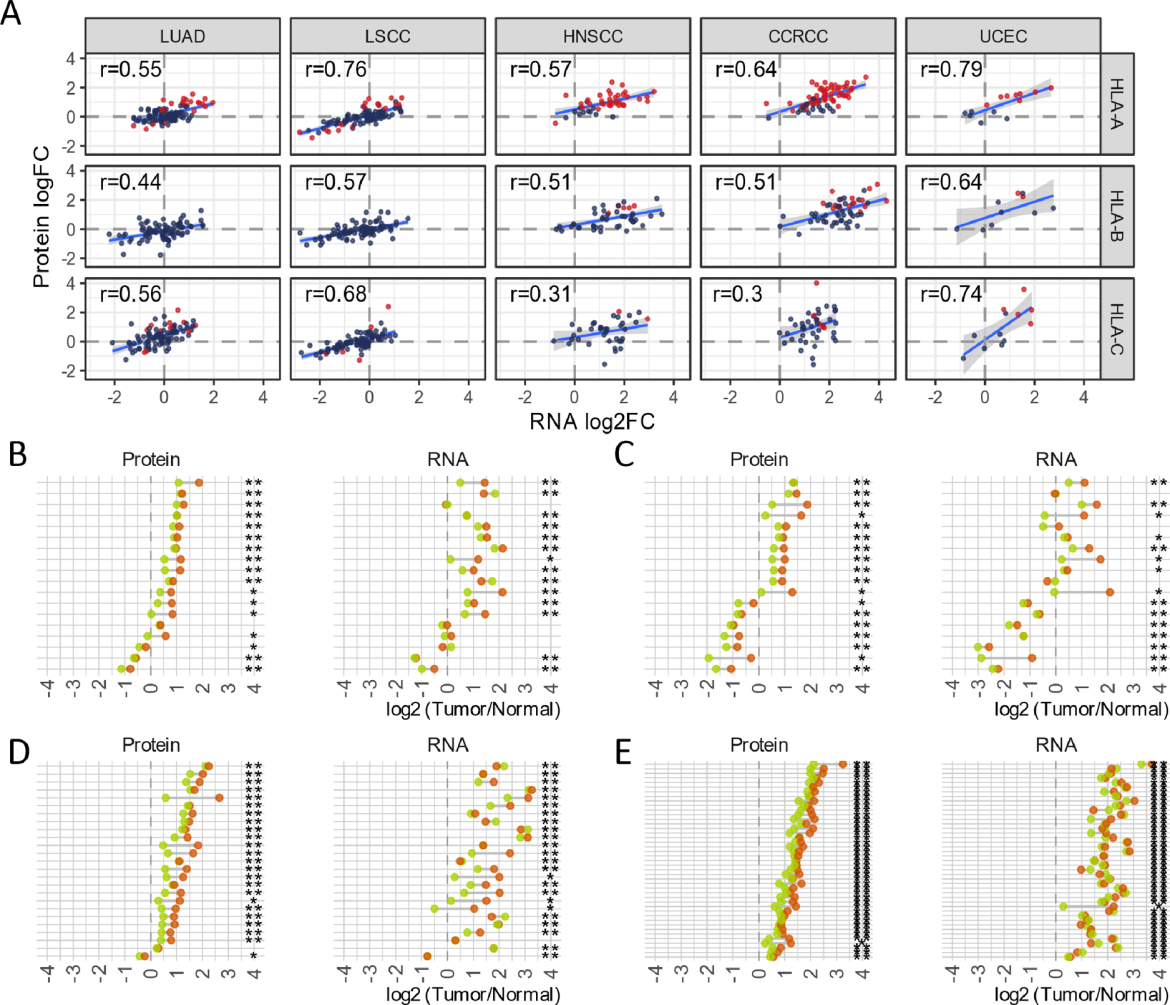
HLA-I is overexpressed in some tumors at both RNA and protein levels. (A) Fold change of HLA-I gene expression in tumor samples over adjacent normal samples from the same patients. The protein expression of HLA-I genes was calculated using only HLA-I gene-specific peptides matching the protein sequences of the sample HLA-I genotypes for that sample. The intensities of such peptides were summed, and then the log2 ratio of such a sum of a sample to the common reference sample was taken to represent the relative protein expression. The log2FC of the protein and RNA is the fold change of tumors over paired adjacent normal samples from the same patient with a relative protein expression or normalized RNA expression, respectively. *r*: Pearson correlation coefficient. Red: significant over- or underexpression in tumor samples compared to adjacent normal samples. Significance is established by a *t* test of all eligible peptides from same samples, comparing tumor with normal samples. (B–E) Allele level tumor/normal expression fold changes in the protein and RNA for the HLA-A gene in heterozygous patients of (B) LUAD, (C) LSCC, (D) HNSCC, and (E) CCRCC. X-asix: log2 tumor/normal fold change of the HLA-A protein (left) and RNA (right). Two colored circles representing two alleles. The protein fold change was calculated for two alleles separately using peptides only matching one of the two alleles excluding ones with a sequence shared by both alleles. Only patients with significant (multitesting adjusted *p* value <0.05) overexpression/underexpression of HLA-A in proteins and that have three or more unique allele-specific peptides in both tumor and normal samples were included in the analysis. Stars: number of alleles that have over 20% overexpression or underexpression in tumor than normal samples on the same allele.

Using sample-specific genotype information, we quantified HLA-I genes at both the RNA and protein level. HLA-I protein expression was largely correlated to RNA expression with Spearman’s rho ranging from 0.5 to 0.8 for HLA-A depending on cancer types (Figure S5). Correlations for HLA-B and HLA-C are lower however, which is likely due to fewer peptides detected, causing a lower quality of protein expression estimation. The level of correlation was in line with our general expectation for protein-RNA correlation.^28^ The expression levels in tumor samples are highly variable on both RNA and protein levels with a 4–10-fold change range, while the dynamic ranges for adjacent normal samples are smaller. Contradictory to our expectation of prevalence of HLA loss in tumors, we observed a prominent high expression of HLA-I genes in tumors when compared with their corresponding adjacent normal samples. This overexpression was observed on both RNA and protein levels and is highly cancer-type-dependent. With quantification from multiple peptides, we used a *t* test to compare protein expression between each of the paired tumor and normal samples (Figure S6). Out of five tumor types with data from adjacent normal samples, almost all HNSCC, CCRCC, and UCEC patients showed an over-twofold expression in tumor samples compared to normal samples. For the HLA-A protein, such differences were statistically significant (multitesting adjusted *p* value <0.05) in 41–63% of sample pairs (Figure 3A). HLA-B and HLA-C also showed the same trend but lacked statistical power due to the low number of peptides. The fold changes between tumor and paired normal samples are also highly consistent on RNA and protein levels (Figure 3A), confirming that the overexpression was largely driven at a transcriptional level. Underexpression of HLA-I in tumors was also observed in some tumors but is mostly limited to LUAD and LSCC patients. It is possible that the overexpression of HLA-I is a response to high levels of antigen peptides produced by rapidly dividing tumor cells, which produce large amounts of misfolded and defected proteins.

We further investigated how overexpression and underexpression in tumors are displayed on two alleles. Because HLA-B and HLA-C genes have a limited number of unique allele-specific peptides available in all data sets, we only inspected peptides from the HLA-A gene. For each of the tumor normal pairs that were heterozygous in HLA-A and showing significant protein over/underexpression (adjusted *p* value <0.05), we compared the abundance of each of two alleles by using allele-specific peptides only if there are over three unique peptides for each allele in both tumor and normal samples. Majority of patients (LUAD: 66.7%, LSCC: 81.8%, HNSCC: 91.7%, and CCRCC: 97.7%) showing significant overall HLA-A protein overexpression also showed over 30% overexpression on both alleles. Most such patients also showed overexpression on both alleles in tumors on the RNA level except for LSCC patients (Figure 3B–E). In a few patients, the tumor-normal fold change was much higher in one allele than the other and their RNA levels also showed a similar trend, suggesting that the allele specific overexpression mostly originated from RNA. Interestingly, a few patients of LUAD and LSCC showed overexpression in proteins on one or both alleles while neither allele showed any overexpression at the RNA level. What kind of post-transcriptional regulation was involved is not clear and still needs more investigation.

### HLA-I Expression Is Positively Associated with Immune Cell Types Universally and Negatively Associated with Endothelial Cells and Neuronal Cells in Some Cancer Types

**Figure 4:**
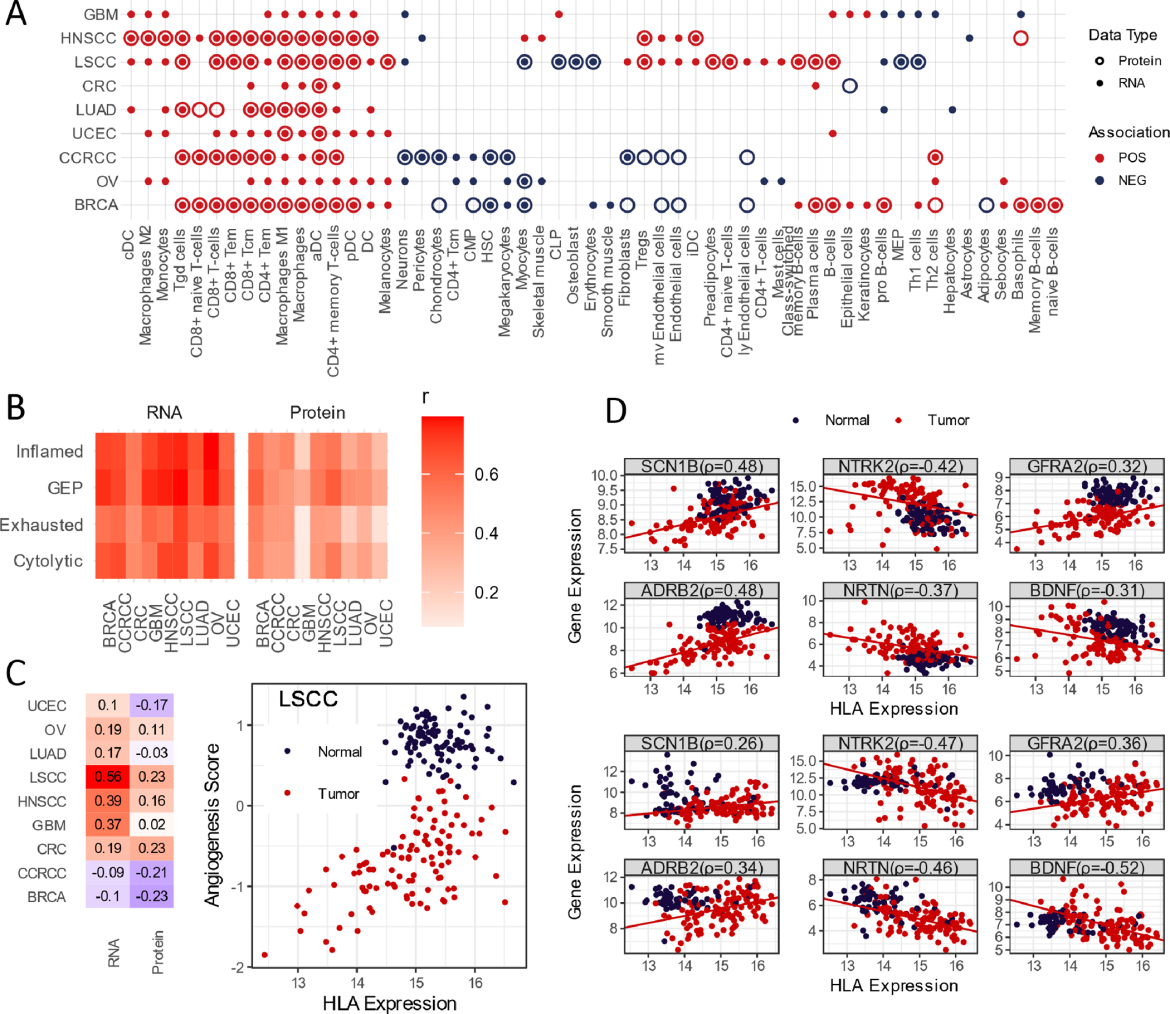
HLA-I expression is positively associated with T-cell infiltration and functions in all cancer types, while the association with angiogenesis and neuronal functions were limited in specific cancers. (A) Association of HLA-I RNA and protein expression with xCell enrichment. Tumor sample cell-type enrichment was determined by xCell analysis. Linear model xCell enrichment score–HLA-I expression (RNA and protein, respectively) was fitted for each of the nine cancer types. HLA-I expression was determined as significantly associated when the BH-adjusted *p* value < 0.05 and, only the significant cell type/cancer was plotted. Red: positive association - coefficient of the HLA-I expression in the linear model > 0; blue: negative association - coefficient < 0. (B) Correlation of T-cell functions with HLA-I RNA and protein expression. Tumors were scored with four different T-cell function signatures using RNA gene expression. For an inflamed, exhausted, and cytolytic score, the mean Z score across all tumor samples from the same cancer types were used as the signature score. For GEP, the weighted linear combination of gene expression was calculated as the signature score, with weights for each member gene provided in ref (30). The HLA-I gene expression was determined by the calculated mean expression of HLA-A, HLA-B, and HLA-C for RNA and protein, respectively. *r*: Pearson correlation coefficient between the signature score and HLA-I expression, labeled on chart. (C) Angiogenesis activity is positively correlated with HLA-I expression only in limited cancer types including LSCC. Angiogenesis scores were calculated as mean Z score of the RNA gene expression of a 43-gene core human primary tumor angiogenesis signature. Left: Pearson correlation between HLA-I expression and angiogenesis score in nine cancer types. Right: scatter plot showing HLA-I expression and angiogenesis score in LSCC tumor and adjacent normal samples. Correlation was observed in tumor samples only. (D) Neuronal genes show a mixed relationship with HLA-I expression in LSCC (upper) and HNSCC (lower) tumors. HLA-I expression was determined as means of HLA-A, HLA-B, and HLA-C RNA expression. Gene expression was represented by RNA expression of neuronal genes. Spearman correlations were calculated using tumor expression data only and labeled on the chart.

To understand how HLA-I levels in tumors are associated with the tumor microenvironment (TME), we applied xCell analysis^21^ on transcriptome data to characterize all tumor samples with different cell type features (Figure S7a) and then identified cell types that are significantly affected by HLA-I RNA and protein expression. Among 64 different cell-type signatures, HLA-I is positively associated with CD8 T cells, CD4 T cells, dendritic cells, macrophages, and B cells in most cancer types, and the associations were observed on both RNA and protein levels (Figure 4A and Figure S7b). Some specialized cell types involved in neurons, blood vessels, muscles, and bone structures are negatively associated with HLA-I but are limited to LSCC, CCRCC, OV, and BRCA. Interestingly, a few progenitor cell-type signatures such as CMP, HSC, CLP and MEP are showing negative association with HLA-I in CCRCC, BRCA, GBM, and LSCC, suggesting the nondifferential nature of the immune component of the HLA-I low tumors from those cancer types.

To further investigate how various tumor-infiltrating T-cell activities are associated with HLA-I, we correlated scores of four gene signatures, representing different T-cell activities in the tumor to both HLA-I RNA and protein expression. All four signatures, including the tumor T-cell inflamed tumor microenvironment,^29^ which is a T-cell inflamed GEP (gene expression profile), are derived from checkpoint inhibitor therapy.^30^ T-cell exhaustion^31^ and T-cell cytolytic activity^32^ are both highly correlated to HLA-I RNA expression, but the correlations with the HLA-I protein level were low in GBM (Figure 4B). GBM is long known to lack infiltrating T-cells, and the tumor induces T-cell exhausting and senescence.^33^ The decoupling T-cell activity with the HLA-I protein is likely yet another aspect of this T-cell dysfunction in GBM.

High tumor mutation burden has been identified as an indicator of immune inflamed tumors and a predictor for response to immune checkpoint inhibitor therapy. Considering the strong signal of T-cell activity in HLA-I high tumors, we investigated the relationship between a non-synonymous mutation count and HLA-I expression. HLA-I expression does not correlate with mutation counts in general (Figure S8a). We observed a moderate positive correlative trend with the HLA-A gene in breast and ovarian cancer but also a slight negative trend in LUAD tumors on the RNA level. Therefore, the neo-antigen from mutation is not likely to be a significant contributor to the strong T-cell activities in HLA-I high tumors.

Endothelial cells are an important component in angiogenesis and play an important role in supporting the gatekeeping role in immune surveillance under normal physiological conditions.^34^ Tumor-associated endothelial cells (TECs) are known to express both MHC-I and MHC-II as antigen-presenting cells but also express immune inhibitory molecules such as PD-L1 and PD-L2.^35^ The complex nature of TEC is also demonstrated in our analysis, showing a divergent relationship with HLA-I: a positive association with HLA-I RNA expression in two squamous cell carcinomas, namely, LSCC and HNSCC, but a negative association with HLA-I protein expression in CCRCC and BRCA. We use a 43-gene core human primary tumor angiogenesis signature^36^ to calculate angiogenesis scores for each tumor and correlate the score with HLA-I expression. Concordant with the xCell analysis result, a moderate negative correlation was observed on the protein level in CCRCC and BRCA and the most striking correlation is the positive correlation with HLA-I RNA expression in LSCC (Figure 4C). This correlation was only observed in tumors but not in normal samples, and the overall angiogenesis and HLA-I expressions are considerably lower in tumors.

We also observed negative association of neuron enrichment with HLA-I, on the RNA level only, in LSCC, GBM, CCRCC, and OV tumors. The trend was also seen in HNSCC but did not reach statistical significance. To identify genes driving the association with HLA-I, we correlate HLA-I expression with a collection of neuronal genes across the cancer types. Some neuronal genes showed strong negative correlation with HLA-I expression, while others are positively correlated. Among the genes most negatively correlated are some neurotrophins and their receptors involved in nerve recruitment and growth, including neurturin (NRTN), BDNF and its receptor TRKB in LSCC and HNSCC (Figure 4D). On the β2 adrenoreceptor (ADRB2) and sodium voltage-gated channel gene (SCN1B), which mediate signaling of neurotransmitters through Gs proteins, are positively correlated with HLA-I in the same tumor types. These results paint a complicated and seemingly contradictory picture of how neuronal activity is involved in the immune component of the tumor microenvironment.

### Senescence and Autophagy Influence HLA-I Protein Levels

**Figure 5:**
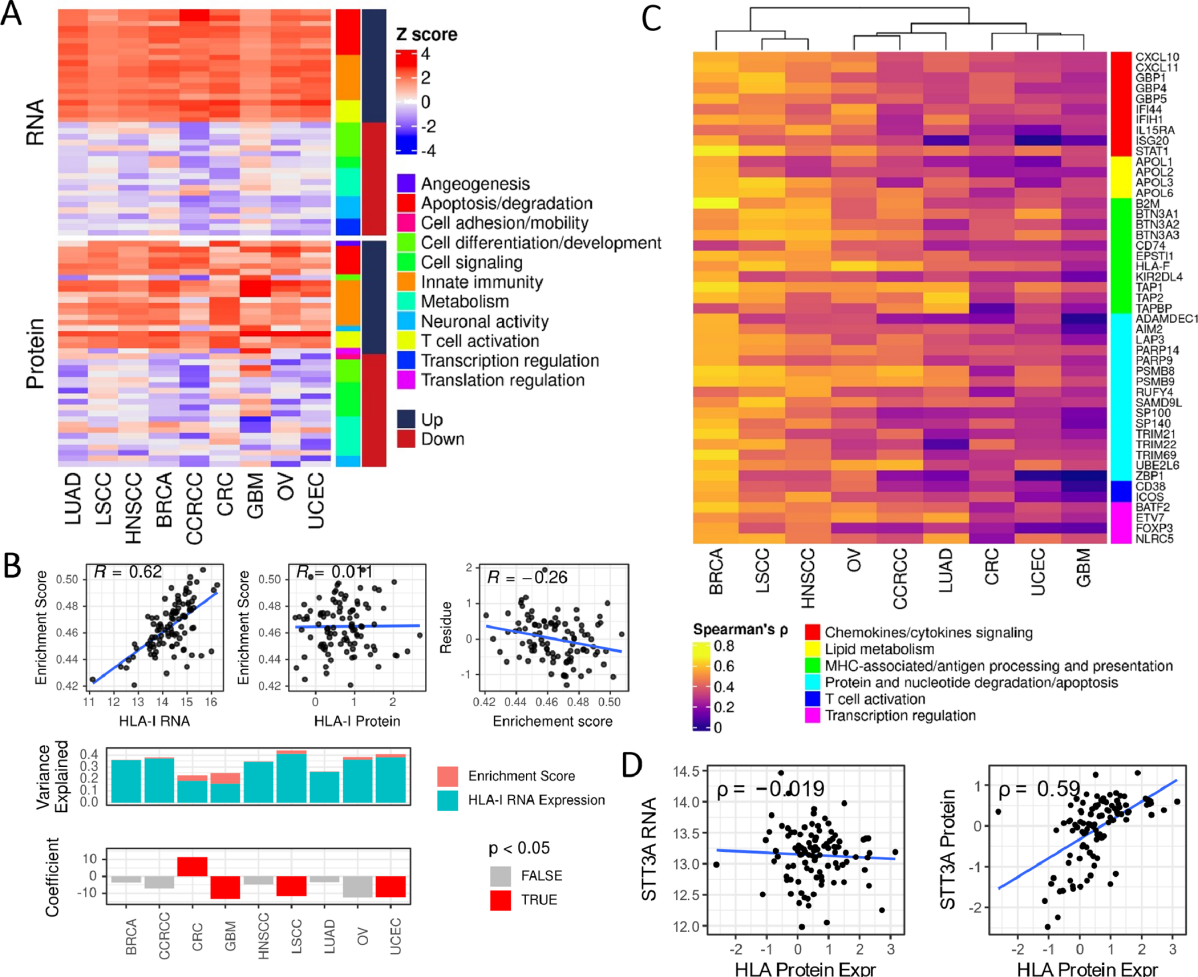
Apoptosis, degradation, and innate immune pathways are universally correlated to HLA-I expression across different cancers. (A) Most correlated pathways with HLA-I RNA and protein expression. The pathway score was determined by SSGSEA (single-sample gene set enrichment) analysis using WikiPathways gene sets and gene expression data of whole transcriptome. Pearson correlations between HLA-I expression (upper: RNA, lower: protein) and enrichment scores were calculated and Z-scored for each of the cancer types. Top: 20 most positively correlated pathways; Bottom: 20 most negatively correlated pathways. Pathways were manually summarized into different categories shown in chart. (B) The “senescence and autophagy in cancer” gene set enrichment is correlated with HLA-I RNA expression but not protein expression in GBM. Upper left: scatter plot of the HLA-I RNA expression and enrichment score showing positive correlation. Upper middle: HLA-I protein expression is not correlated with the enrichment score. Upper right: the residue of the linear model (HLA-I protein expression–HLA-I RNA expression) trended negatively with the enrichment score. Bottom: effect of the “senescence and autophagy in cancer” gene set enrichment on the HLA-I protein expression across nine cancer types. The portion of variance explained was determined by the *r* squared of linear models of HLA-I protein expression–HLA-I RNA expression + enrichment score. The linear model was fit for each of the cancer types. Coefficients of the enrichment score variable was plotted in the lower panel. Negative coefficients in GBM, LSCC, and UCEC indicated that higher enrichment in senescence and autophagy is associated with low HLA-I protein expression. Only in GBM, the effect of enrichment contributes substantially to explain the variance and is negatively associated with the HLA-I protein level. (C) Genes most correlated to HLA-I protein levels. Spearman’s *r* was calculated for the correlation between the RNA expressions of gene and the HLA-I protein expression. Only expression data from tumor samples were used. Genes were manually categorized into different groups based on their biological functions. (D) Correlation between HLA-I and STT3A in UCEC. HLA-I protein expression was determined by taking the average of HLA-A, B, and C expression. Spearman’s *r'*s were calculated between STT3A RNA expression (left) and protein expression (right) in UCEC. Only tumor samples were used in the analysis.

To identify biological processes and pathways associated with the HLA-I level, we performed single-sample gene set enrichment analysis (SSGSEA) on all tumor samples against mSigDB wikipathway collections and then correlate SSGSEA scores with both HLA-I RNA and protein expression. As we expected, pathways involving immune response, including T-cell cytolysis activities, showed the highest positive correlations including interferon signaling, toll-like receptors, and T-cell activations. Also highly correlated are apoptosis and DNA/protein degradation pathways (Figure 5A). Positively correlated pathways were highly concordant among different cancer types and were observed on both HLA-I RNA and protein expression. There are fewer pathways negatively correlated with HLA-I levels, and most such correlations were limited to certain cancer types. In general, pathways in cell differentiation/development, carbohydrate and lipid metabolism, and a few kinase signaling pathways and neuronal activities are the most common ones. The most prominent negative correlation was observed in CCRCC, including the WNT pathway, endothelial cells' pathway, and pathways involving neural functions including synaptic signaling.

We noticed that the signature of “senescence and autophagy in cancer”, which includes all major autophagy genes, is highly correlated with HLA-I RNA expression in GBM (*r* = 0.62), but such a relationship was not observed on the HLA-I protein level (*r* = 0.01) (Figure 5B). Previous study showed that HLA-I molecules are actively removed and digested through autophagy machinery in pancreatic cancer cells, preventing tumor cells from being targeted by cytotoxic T cells. We suspect that this mechanism could be adopted by GBM as well. We hypothesized that higher levels of autophagy in HLA-I high cells results in a dampened level of HLA-I proteins, which explained the lack of correlation with protein expression. To quantify the influence of autophagy on HLA-I expression, we modeled HLA-I protein expression with HLA-I RNA expression as a variable. The residues, representing how protein expression deviates from what is predicted solely from the RNA level, trended negatively with the autophagy signature scores. When we added the autophagy signature score as an additional variable to the linear model, the variance explained, which is represented by the *R* squared of the model fit, increased from 15.8 to 24.8% in GBM. This phenomenon was limited to GBM alone with a similar trend but a much lower signal from UCEC tumors. The link between autophagy and HLA-I needs more investigation from a wider scope of cancer types.

### HLA-I Protein Expression Influences Gene Expression with Immune Response and Cell Death Processes

To understand what transcriptional programs either influence or are implicated by the HLA-I protein level, we looked for the genes with their RNA expression highly correlated to the HLA-I protein across all cancer types. A limited number of genes were identified, and the highest correlation (Spearman’s ρ) is 0.69. Majority of the genes were identified in BRCA, LSCC, and HNSCC, and almost no genes are highly correlated in GBM and CRC (Figure 5C and Table 4). Other than HLA-I molecules themselves, all genes are involved in a few processes directly associated with HLA-I functions in both adaptive and innate immune systems, including T-cell activation/suppression, antigen presentation, cytokine/chemokine pathways, nucleotide and protein degradation, and cell death processes. PSMB8 and PSMB9 are the most universally correlated genes across different cancer types, suggesting highly coordinated proteasome activity with the HLA-I level. By producing peptide antigen, the proteosome may stabilize HLA-I molecules by providing them with peptides to bind. To further illustrate the relationship between HLA-I and proteasome genes, we correlate HLA-I with the expression of various components of the proteasome complex, including the 20S core particle, 19S regulatory particle, 11S regulator, assembling chaperones, and proteasome-interacting proteins (PIPs) on both RNA and protein level. Besides PSMB8, PSMB9, and PSMB10, parts of the 20S core particle and 11S regulator genes PSME1 and PSME2 are also highly correlated to HLA-I on both RNA and protein levels across all cancer types (Figure S8b).

**Table 4:** List of Top Correlated Genes to the HLA-I Protein at RNA and Protein Level^a^

function	both	RNA	protein
chemokines/cytokines signaling	STAT1 GBP1 ISG20	GBP4 IL15RA IFIH1 GBP5 CXCL10 CXCL11 IFI44	DOCK2 GBP2
lipid metabolism	APOL3 APOL6	APOL1 APOL2	ABHD16A ALG6
MHC-associated/antigen processing and presentation	TAP1 TAP2 TAPBP BTN3A1 BTN3A3 BTN3A2 HLA-F EPSTI1	CD74 B2M KIR2DL4	
protein and nucleotide degradation/apoptosis	TRIM22 PSMB8 PSMB9 UBE2L6 PARP14	SAMD9L RUFY4 LAP3 SP100 SP140 ZBP1 TRIM21 ADAMDEC1 PARP9 AIM2 TRIM69	PSMB10 PML CYBA CYBB OAS2 PSME2 PML SYVN1
T-cell activity		CD38 ICOS	CD247 CD48 PTPRC
transcription regulation		ETV7 NLRC5 FOXP3 BATF2	CLCC1 REEP5 c5orf15
protein glycosylation and transport			LMAN2 RNF213 DDOST LMAN1 RFT1 SEC11C STT3A

a Top correlated genes were identified using Spearman’s rho of >0.55 to HLA-I protein expression, which was determined by the mean log ratio of HLA-A, B, and C protein levels in at least one study excluding HLA-I genes. The correlations were calculated for both RNA expression and protein expression for all genes. The overlap of the two lists of top correlated genes is in the “both” column. Genes in the top correlated gene list on the RNA level or protein level only are in respective columns. The genes were grouped by their functions manually.

We also identified co-expressed proteins with HLA-I proteins. Only 18 genes showed a high correlation on the RNA level. However, the co-expressed proteins fall in similar categories especially for genes involved in protein and nucleotide degradation and apoptosis processes. However, a group of genes functioning in protein glycosylation and transport showed co-expression exclusively on the protein level (Figure 5D). Among these proteins are STT3A and RFT1, and both are ER-associated and part of N-linked protein glycosylation machinery.^37^ STT3A is a subunit of N-glycosyltransferase, and RFT1 is believed to be part of a flippase assembly, transferring a precursor oligosaccharide across the ER membrane to be utilized by glycosyltransferase.^38^ The protein expressions of STT3A and RFT1 are highly correlated in most cancer types, and both proteins showed high correlation to the HLA-I protein level in several cancer types (Figure S8c). However, the RNA expression of these two proteins was not correlated with the HLA-I protein level with such a discrepancy most obvious in UCEC (Figure 5D), LUAD, and LSCC tumors. These results suggest a link between protein glycosylation and the immune features of cancer. Indeed, it has been shown that STT3 was transcriptionally induced by epithelial–mesenchymal transition (EMT) through β-catenin, and STT3-dependent PD-L1 N-glycosylation stabilizes and upregulates PD-L1 in tumor cells.^39^ The mechanism of how HLA-I is involved has yet to be discovered.

## Discussion

Accurate HLA-I expression assessment is imperative for understanding how tumor cells interact and influence their surrounding microenvironments to escape immune surveillance. Although HLA-I loss can be successfully estimated at the genomic level by DNaseq results, the mRNA and protein expression profile of HLA-I are not widely studied. In cancer, it is not uncommon that genomic events, such as the DNA copy number gain and loss, have diverse outcomes on mRNA and protein expression, deviating greatly from the prediction from the number of DNA copies. The high variability in HLA-I sequences further complicates the quantification. In RNAseq data analysis, commonly used sequence references often include a very limited number of HLA-I sequences. Sequencing reads from the variable region of the HLA-I gene may not be properly aligned if the genotype is not recorded in the reference. On the other hand, reference sequences not matching the sample genotype are likely to interfere with the correct identification of the source of the reads. The advantage of using sample genotype-specific references to quantify mRNA is twofold: it provides correct reference sequences to match the HLA-I reads and eliminates the noise nonrelevant reference sequences introduced by erroneous alignments. In MS proteomic assays, the protein identity is determined by matching the spectra of detected peptides with all theoretical masses produced from reference proteome sequences. Adding personalized HLA-I entries enables the identification of HLA-I peptides that would otherwise be missed from standard references. Unlike RNAseq, the portion of protein sequences captured by mass-spec-based methods is very low. In practice, six unique peptides per 1000 amino acids of protein length was considered as the minimum for protein quantification in multiple CPTAC studies.^16,40^ Therefore, detecting additional HLA-I peptides is especially valuable to improve quantification. What is more informative are the peptides with sequences differentiating the two alleles of the same HLA-I gene, which provide identification and quantification of proteins produced by each of the two alleles. Our study validated the value of MS-based proteomic data in HLA-I quantification even when the assays were not HLA-I targeted by design.

Contradictory to previous belief, we demonstrated that neither an HLA-I loss of heterozygosity nor expression downregulation in the tumor is a high-prevalence event. Instead, we observed overexpression in tumor cells compared to the surrounding normal tissue in several cancer types. This overexpression was evident in RNA expression and confirmed at the protein level especially in CCRCC and HNSCC, where almost all tumors overexpressed HLA-I. For CCRCC, immunohistochemistry study using an anti-HLA-ABC antibody corroborated this result, showing HLA-I expression in 95.6% of the tumors versus only 12.9% of normal tissues.^41^ However, we must recognize the limitations of the RNA and protein expression data we use when comparing tumors with normal samples directly. The cell components and origin of the tumor can be very different from adjacent normal samples, and gene expression can be highly dependent on cell types. Cell-type analysis of CCRCC tumor samples revealed a lack of epithelial signature when compared to the adjacent normal, which is different from other tumor types. Using the single-cell RNAseq data from Tabula Sapiens Consortium (https://tabula-sapiens-portal.ds.czbiohub.org), we found that HLA-I gene expression is relatively low in kidney epithelial cells, which comprises over 85% of the cells in kidney samples from normal human donors. This is not true for NHSCC tumors though since the epithelial signature in tumors is at a similar level compared to adjacent normal samples. In general, HLA-I genes are expressed highly in endothelial cells and immune cells including T cells, B cells, and dendritic cells. We did find a portion of CCRCC tumors displaying a high endothelial cell signature. The effect of the cellularity discrepancy between tumor and normal samples has been a major limitation for any analysis in an attempt to make a tumor–normal comparison. The level and direction of bias introduced by this limitation are highly dependent on the cancer type and not easy to quantify. Assays on the single-cell level will be helpful to address such issues. On the other hand, bulk tumor sample assays lose spatial information on HLA-I RNA and protein sources. Even when overexpression is observed in tumors, it is very possible most of the HLA-I molecules are from a fraction of tumor or stromal cells. Furthermore, we were not able to determine if the HLA-I molecules in tumors are functioning proteins and located at the cell membrane presenting tumor peptides. Still, pathway analysis showed a high correlation between immune activities, including IFNγ signaling, and the HLA-I expression level. This suggested that HLA-I loss or downregulation may not be a universal and necessary feature of all tumors. Other mechanisms independent of HLA-I are responsible for escaping T-cell responses.

Results from correlative analysis on HLA-I expression reveal cellular activities mostly expected from orthodox HLA-I functions including antigen presentation, cytokine/chemokine signaling, protein degradation, and cell death. However, the analysis also highlighted processes not previously linked to HLA-I, such as cancer-type dependent angiogenesis activity and high co-expression of glycosylation genes. Although the data from this study are not sufficient to provide a hypothesis to explain such links, these findings suggested a possible wider scope of HLA-I involvement in tumor biology beyond their antigen-presenting activities. Especially interesting is that HLA-I and glycosylation genes are highly concordant on the protein level, suggesting a direct interaction of HLA-I with glycosylation mechanisms. Glycosylation is extensively involved in cancer with altered functions impacting tumor cell–cell adhesion, cell–matrix interaction and signaling, immune surveillance, and tumor metabolism.^42^ The fact that antigens presented on tumor cells are often highly glycosylated with specific carbohydrates added the possibility of a cooperation between an HLA-I peptide complex and glycosylation in cancer. We believe the findings from our study provide a new perspective worth further perusing to better understand HLA-I in cancer.

## Data Availability

All data used in this study are publicly available for download. CPTAC-2 and CPTAC-3 RNAseq bam files were downloaded from the Genomic Data Commons Data Portal (https://portal.gdc.cancer.gov). Proteome mass spectrum files were downloaded from Proteomic Data Commons (https://proteomic.datacommons.cancer.gov).

## Supporting Information Available

RNA expression difference of HLA-I between two alleles in heterozygous tumors; top 8 allele frequencies of the most prevalent phenotypes of HLA-I genes in CPTAC cancers; HLA-I peptides identified in CPTAC proteomic data; shared and allele-specific HLA-I peptides in CPTAC samples; correlation of HLA-I RNA and protein expression in tumor samples; comparing HLA-I protein expression between paired tumor and normal samples; cell-type enrichment of tumor and normal samples; and correlation between HLA-I and proteasome genes and mutation counts (PDF)
All confidently identified peptides in each of CPTAC studies (XLSX)
All proteins identified from CPTAC MS studies (XLSX)
HLA-I gene peptides confidently identified from CPTAC MS studies (XLSX)

## Related Paper

This work was supported by the grant no. U24CA210972 from the National Cancer Institute’s Clinical Proteomic Tumor Analysis Consortium and the S21–167 contract from Leidos Biomedical Research.

## Abbreviations

AF: allele frequency
B2M: b2-microglobulin
BRCA: breast cancer
CCRCC: clear cell renal-cell carcinoma
CGC: Cancer Gene Census
CPTAC: Clinical Proteomic Tumor Analysis Consortium
CRC: colorectal cancer
DDA: data-dependent acquisition
EMT: epithelial–mesenchymal transition
GBM: glioblastoma
IMGT/HLA: immunogenetics/hla database
HNSCC: head and neck squamous-cell carcinoma
ICB: immune checkpoint blockade
LOH: loss of heterozygosity
LSCC: lung squamous-cell carcinoma
LUAD: lung adenocarcinoma
MAF: minor allele frequency
MS: mass spectrometry
OV: ovarian high-grade serous carcinomas
PIP: proteasome-interacting proteins
SSGSEA: single-sample gene set enrichment analysis
T-DBG: de Bruijn graph
TEC: tumor-associated endothelial cells
TME: tumor microenvironment
TMT: isobaric tandem mass tags
UCEC: uterine corpus endometrial carcinoma
VST: variance stabilizing transformation
WMDA: World Marrow Donor Association
