# Supplementary material for: Proteogenomics Reveal the Overexpression of HLA-I in Cancer
Source: J Proteome Res. 2023 Oct 19;22(11):3625–39. doi: 10.1021/acs.jproteome.3c00491 (PMC10629274; doi:10.1021/acs.jproteome.3c00491)
Supplement: Supplementary file 1 — pr3c00491_si_001.pdf [file pr3c00491_si_001.pdf]

Proteogenomics reveal over-expression of HLA-I in cancer

Authors:

Ying Wang and David Fenyö

Supplemental Figures

Figure S1 RNA expression difference of HLA-I between two alleles in heterozygous tumors

Figure S2 Top 8 allele frequency of most prevalence phenotypes of HLA-I genes in CPTAC cancers

Figure S3 HLA-I peptides identified in CPTAC proteomic data

Figure S4 Shared and allele specific HLA-I peptides in CPTAC samples

Figure S5 Correlation of HLA-I RNA and protein expression in tumor samples

Figure S6 Comparing HLA-I protein expression between paired tumor and normal samples

Figure S7 Cell type enrichment of tumor and normal samples

Figure S8 Correlation between HLA-I and proteasome genes and mutation counts

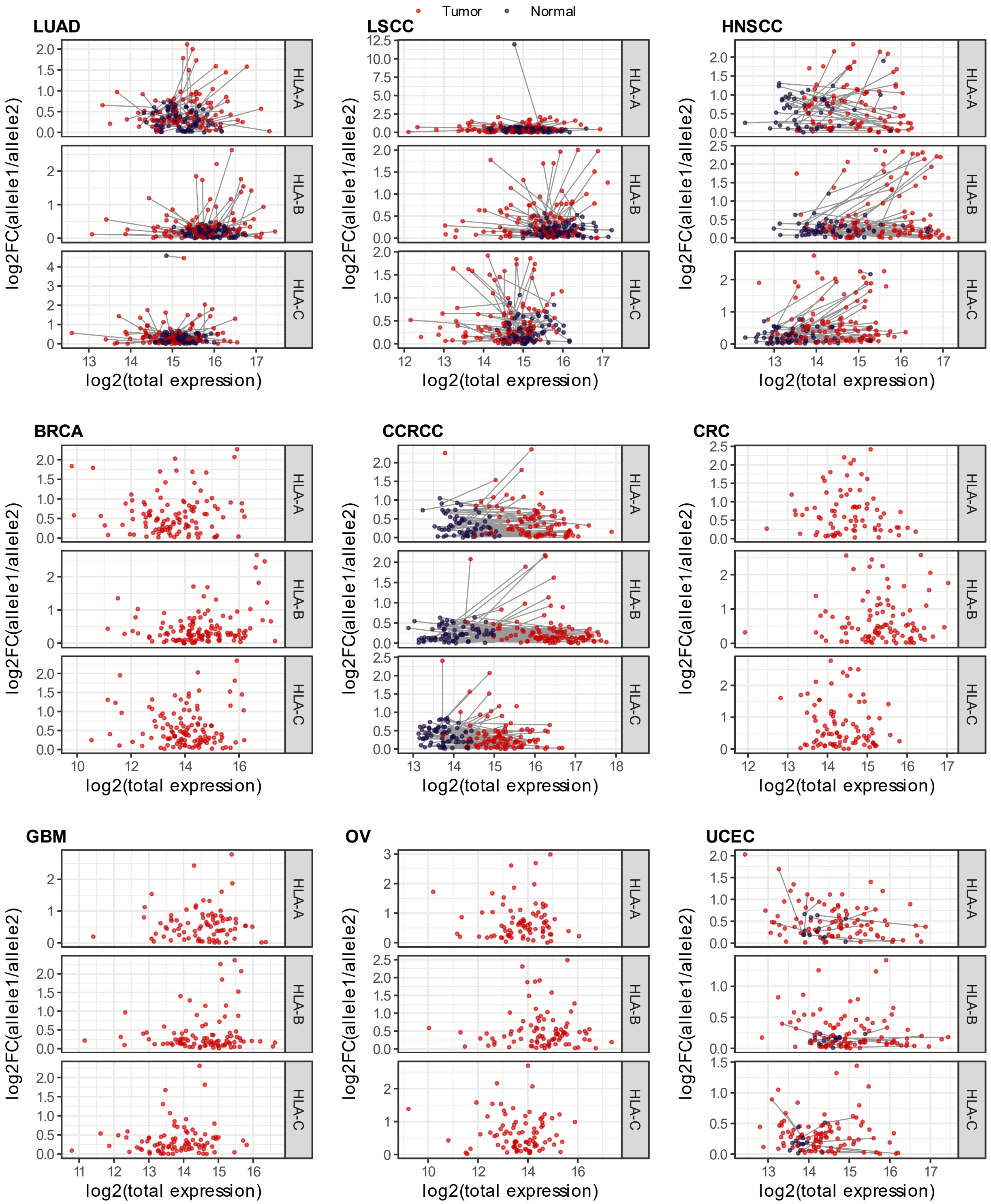
Tumor
Normal
LUAD
LSCC
HNSCC
BRCA
CCRCC
CRC
GBM
OV
UCEC
HLA-A
HLA-B
HLA-C
log2FC(allele1/allele2)
log2(total expression)

Figure S1 RNA expression difference of HLA-I between two alleles in heterozygous tumors

Only samples HLA-I genotyped as heterozygous for a specific HLA-I gene were shown. Total expression of HLA-A, HLA-B and HLA-C was calculated by taking log sum of the VST normalized gene counts from both alleles. Log2FC(allele1/allele2) is the log2 transformed ratio between the normalized expressions of the allele with higher expression and the allele with lower expression. Grey lines connected the tumor and adjacent normal samples from the same patients.

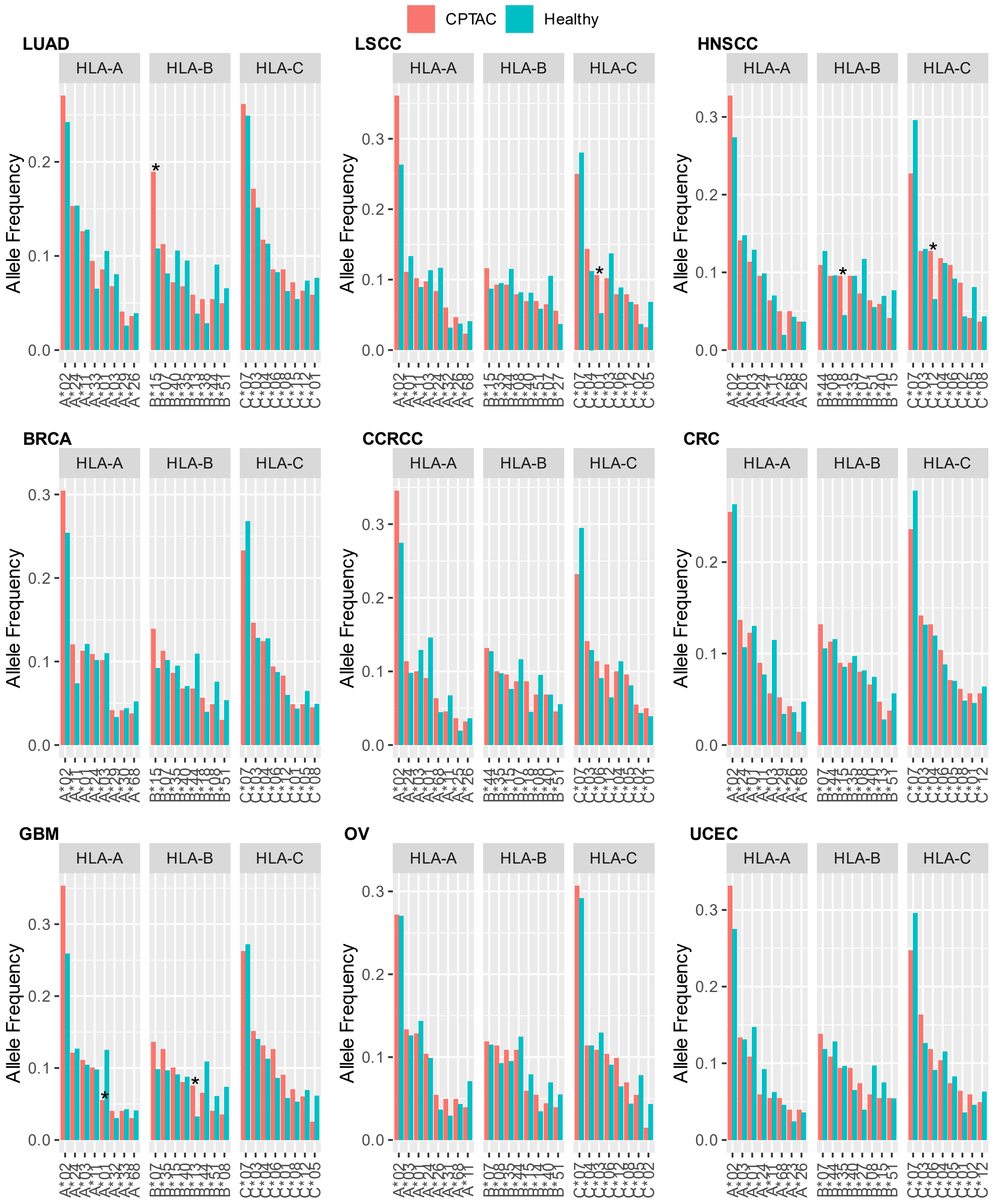
CPTAC
Healthy
LUAD
LSCC
HNSCC
BRCA
CCRCC
CRC
GBM
OV
UCEC
HLA-A
HLA-B
HLA-C
Allele Frequency

Figure S2 Top 8 allele frequency of most prevalence phenotypes of HLA-I genes in CPTAC cancers

HLA-I allele frequency (AF) was calculated using HLA-I genotype results of both alleles for each of the samples in a CPTAC study. Only the most prevalence 8 allele groups in each of the study were shown. The expected AF for healthy population was calculated separately for each of the CPTAC study according to the ethnic composition of the study objects. HLA-I AF of allele groups for each of the ethnic group were calculated using data from Allele Frequency Net Database. P-values for over or under representation for each allele group is estimated using Poisson binomial distribution and adjusted for multi-testing with Benjamini-Hochberg procedure. Allele groups with adjust p values < 0.05 and the difference between the AF in CPTAC study and the expected AF in healthy population is over 50% of expected AF are considered as significantly over/under represented and labeled with * on the plot.

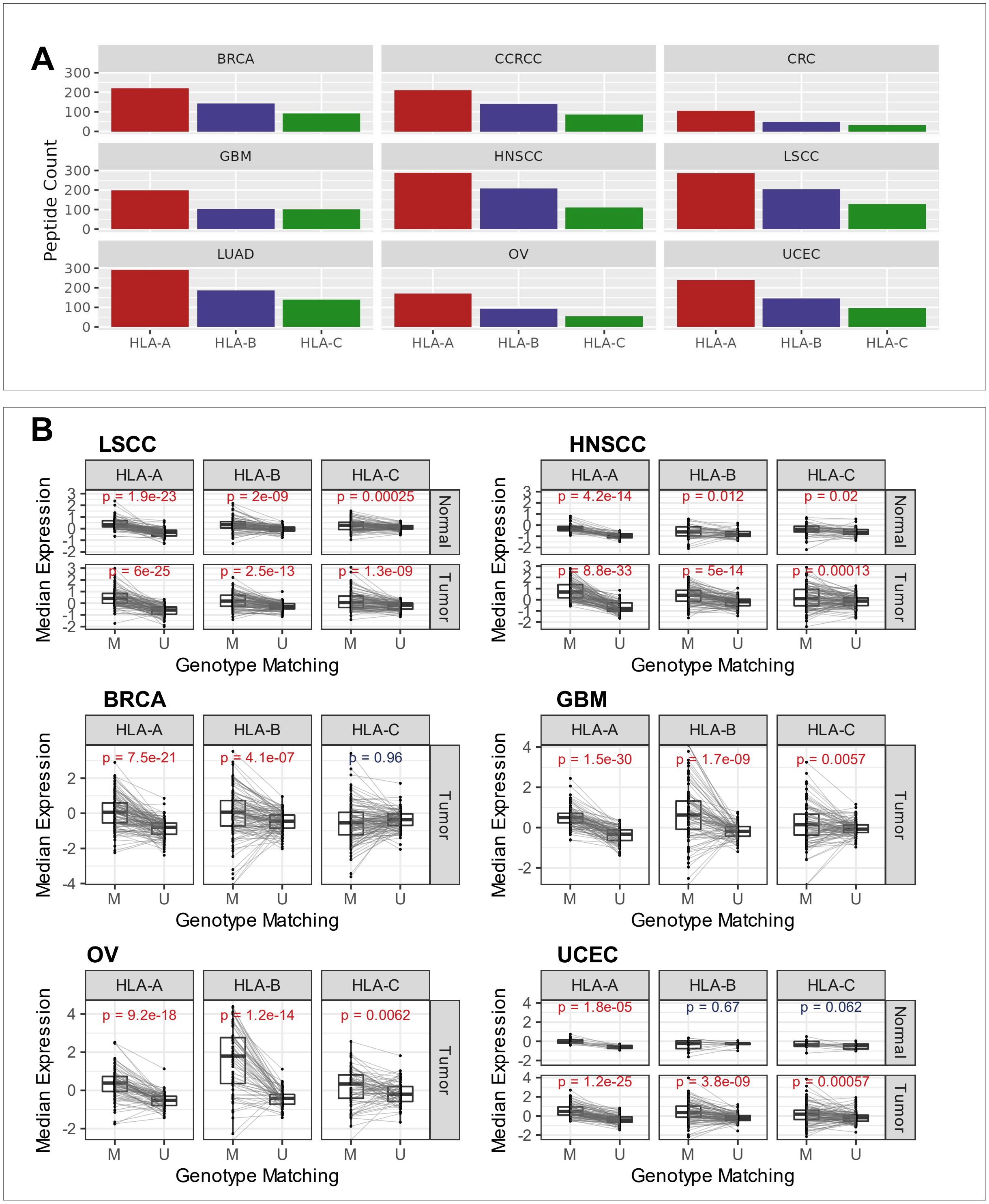
A
Peptide Count
BRCA
CCRCC
CRC
GBM
HNSCC
LSCC
LUAD
OV
UCEC
HLA-A
HLA-B
HLA-C
B
LSCC
HNSCC
BRCA
GBM
OV
UCEC
Median Expression
Genotype Matching
Normal
Tumor
M
U
p = 1.9e-23
p = 2e-09
p = 0.00025
p = 6e-25
p = 2.5e-13
p = 1.3e-09
p = 4.2e-14
p = 0.012
p = 0.02
p = 8.8e-33
p = 5e-14
p = 0.00013
p = 7.5e-21
p = 4.1e-07
p = 0.96
p = 1.5e-30
p = 1.7e-09
p = 0.0057
p = 9.2e-18
p = 1.2e-14
p = 0.0062
p = 1.8e-05
p = 0.67
p = 0.062
p = 1.2e-25
p = 3.8e-09
p = 0.00057

Figure S3 HLA-I peptides identified in CPTAC proteomic data

(A) Number of unique HLA-I peptides identified in CPTAC cancers

Only peptides longer than 6 residues and identified with high confidence (p-value < 0.001) were counted. Peptides with same sequences but different modifications were consolidated as one unique peptide. The peptide with sequences shared among any two or three HLA-I genes or any other genes were removed from counting and expression estimation.

(B) Median expression of genotype matched and unmatched peptides in CPTAC cancers

For each HLA-I gene specific peptide identified in a sample, it was labeled as HLA-I genotype-matched (M) if the sequences can be derived from the protein sequences of the HLA-I genotypes of the sample, otherwise the peptide was labeled as un-matched(U). Expression of the peptide is estimated by taking the log2 transformed ratio of the signal intensity of the sample over the pooled common control sample and then normalized by median of whole proteome of the sample. Median of peptide expression above was calculated for M peptides and U peptides for each of the sample and plotted at y axis, with grey line connecting the two types of peptides from the same sample.

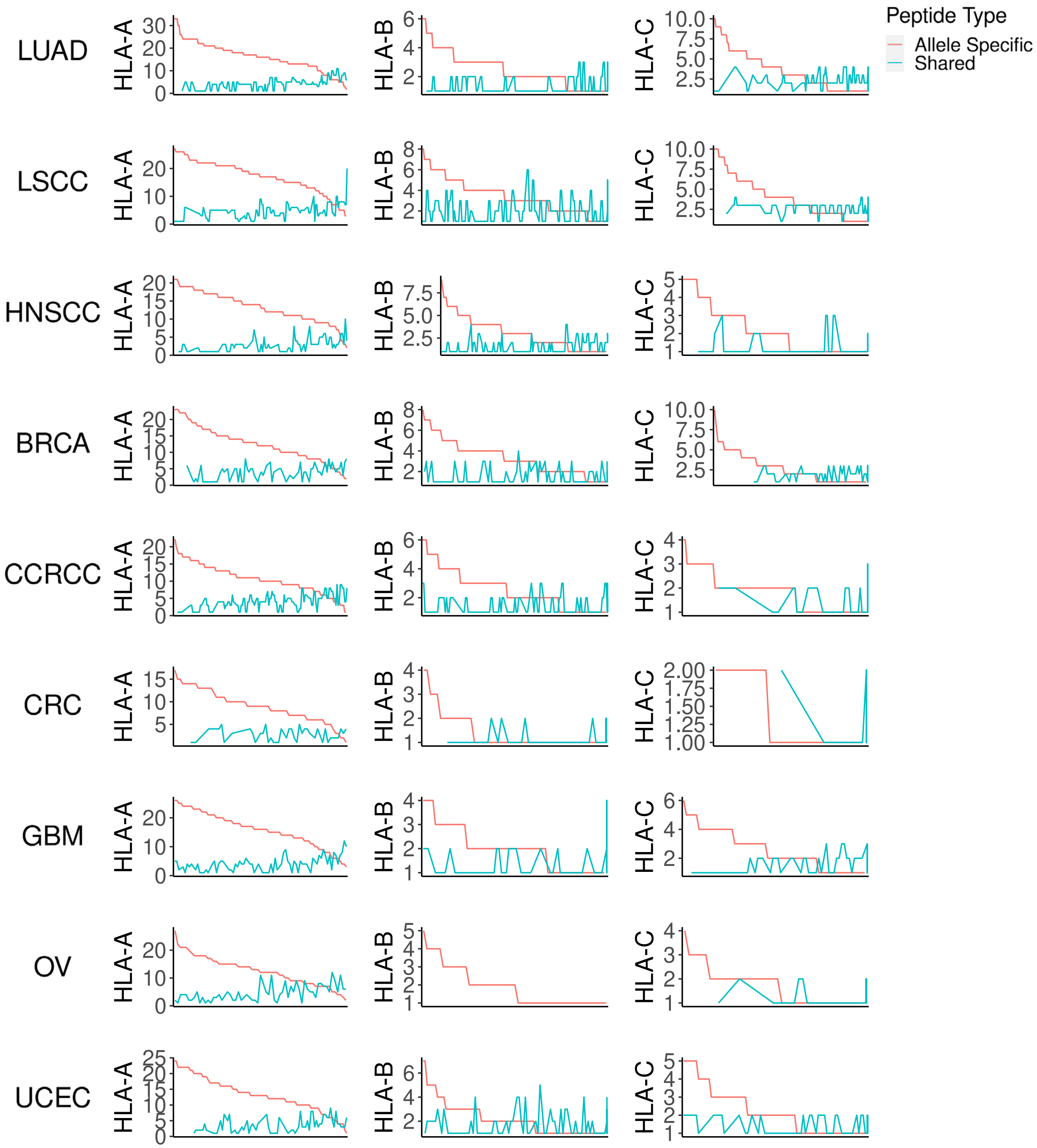
Peptide Type
Allele Specific
Shared
LUAD
LSCC
HNSCC
BRCA
CCRCC
CRC
GBM
OV
UCEC
HLA-A
HLA-B
HLA-C

Figure S4 Shared and allele specific HLA-I peptides in CPTAC samples

For each of the heterozygous samples for a specific HLA-I gene, unique HLA-I gene specific peptides with sequences matching the sample HLA-I genotype were identified and counted. They were then categorized as “allele-specific” (red line) if the sequences can only derive from one of the alleles, or as “shared” (blue line) if the sequence can derived from both alleles. X-axis are the samples ordered by the “allele-specific” peptide counts from largest (left) to smallest (right).

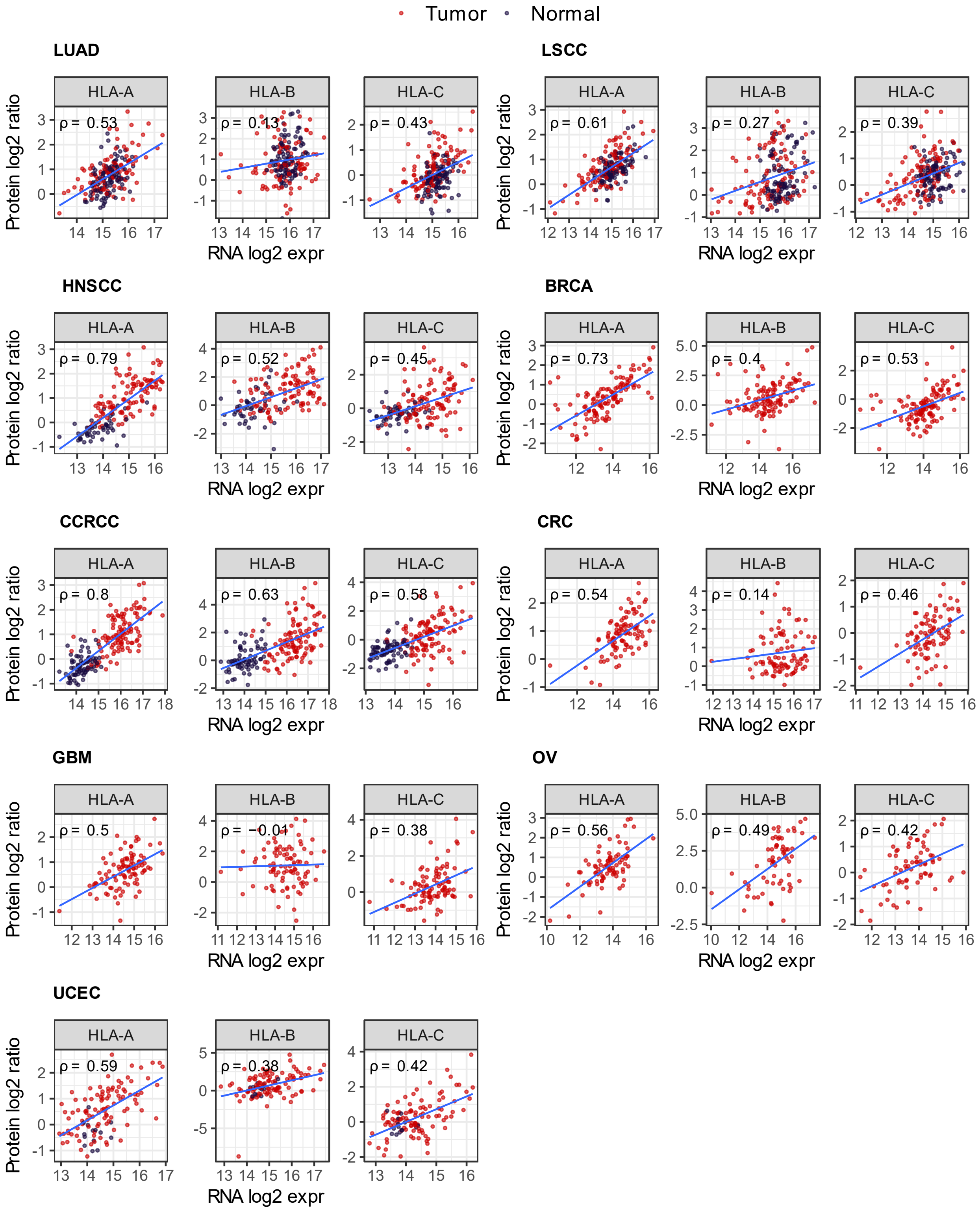
Tumor
Normal
LUAD
LSCC
HNSCC
BRCA
CCRCC
CRC
GBM
OV
UCEC
HLA-A
HLA-B
HLA-C
Protein log2 ratio
RNA log2 expr
ρ = 0.53
ρ = 0.13
ρ = 0.43
ρ = 0.61
ρ = 0.27
ρ = 0.39
ρ = 0.79
ρ = 0.52
ρ = 0.45
ρ = 0.73
ρ = 0.4
ρ = 0.53
ρ = 0.8
ρ = 0.63
ρ = 0.58
ρ = 0.54
ρ = 0.14
ρ = 0.46
ρ = 0.5
ρ = −0.01
ρ = 0.38
ρ = 0.56
ρ = 0.49
ρ = 0.42
ρ = 0.59
ρ = 0.38
ρ = 0.42

Figure S5 Correlation of HLA-I RNA and protein expression in tumor samples

HLA-I RNA expression (x-axis) was estimated by taking log sum of VST normalized counts for both HLA-I alleles if the sample is heterozygous for that HLA-I gene, or normalized counts if homozygous. Protein expression (y-axis) was estimated by the median of expression values of all HLA-I gene specific peptides matching the HLA-I genotype of the sample for each HLA-I genes and then taking the mean of the three genes. The expression values of such HLA-I peptides were determined by taking the log2 transformed signal ratio between the sample and the pooled common control sample.

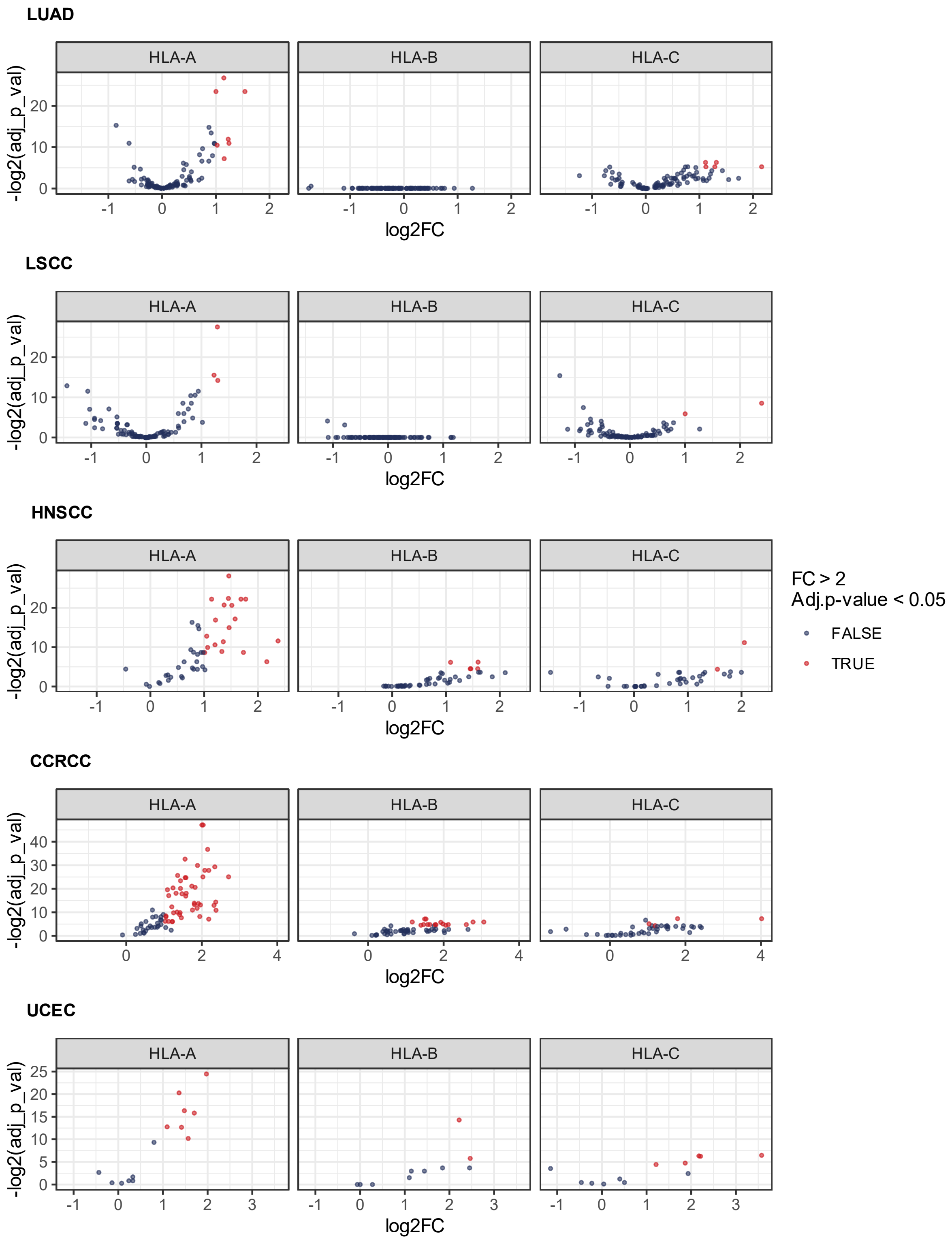
LUAD
LSCC
HNSCC
CCRCC
UCEC
HLA-A
HLA-B
HLA-C
-log2(adj_p_val)
log2FC
FC > 2
Adj.p-value < 0.05
FALSE
TRUE

Figure S6 Comparing HLA-I protein expression between paired tumor and normal samples

For each of the samples in tumor-normal pairs and each of the HLA-I genes, peptides specific to the HLA-I gene and matching the sample HLA-I genotype were selected and used for the analysis. The expression value of each of such peptides was determined by the log2 ratio between sample of pooled common control sample. Each sample has multiple such peptides, therefore provided multiple peptide expression values for the same HLA-I gene. Unpaired t-test was used to compare the means of such peptide expression values between the tumor and normal sample pairs from the same patient. Patients with fewer than three HLA-I gene specific and genotype matching peptides in either tumor or normal samples were removed from the analysis. p-values of the t-test was adjusted for multi-testing with Benjamini-Hochberg procedure.

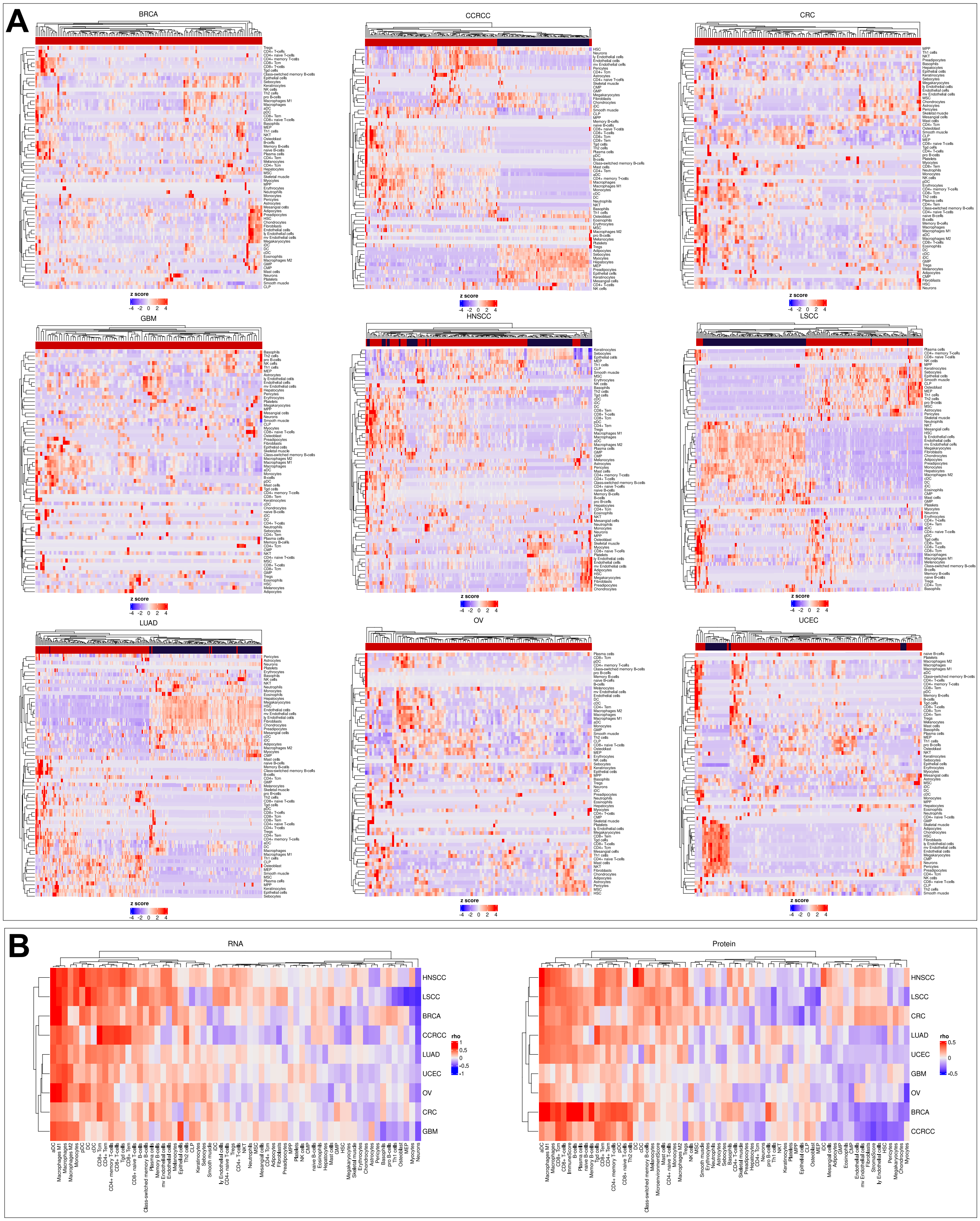
A
BRCA
CCRCC
CRC
GBM
HNSCC
LSCC
LUAD
OV
UCEC
z score
-4 -2 0 2 4
B
RNA
HNSCC
LSCC
BRCA
CCRCC
LUAD
UCEC
OV
CRC
GBM
rho
1
0.5
0
-0.5
-1
Protein
HNSCC
LSCC
CRC
LUAD
UCEC
GBM
OV
BRCA
CCRCC
rho
0.5
0
-0.5

Figure S7 Cell type enrichment of tumor and normal samples

(A) Heatmap of xCell scores of CPTAC samples

Gene expression from transcriptome RNAseq data for each of the CPTAC studies were used in xCell analysis. The xCell scores for 64 cell types were z-scored across all samples in the study for each of the cell type. Sample type indicated in heatmap top annotation bar. Red: tumor; blue: adjacent normal. Samples are clustered and ordered by the xCell scores. For studies with tumor normal sample pairs, samples are mostly clustered by sample type. Patterns of cell type scores varied across the cancer types and highly dependent on the cell components of tissue origin.

(B) Correlation between xCell score and HLA-I RNA and protein expression

Only tumor samples were used in the analysis. xCell scores were correlated with HLA-I RNA expression (left) and protein expression (right) using Spearman's rho. HLA-I expression was determined by the mean of HLA-A, HLA-B and HLA-C genes.

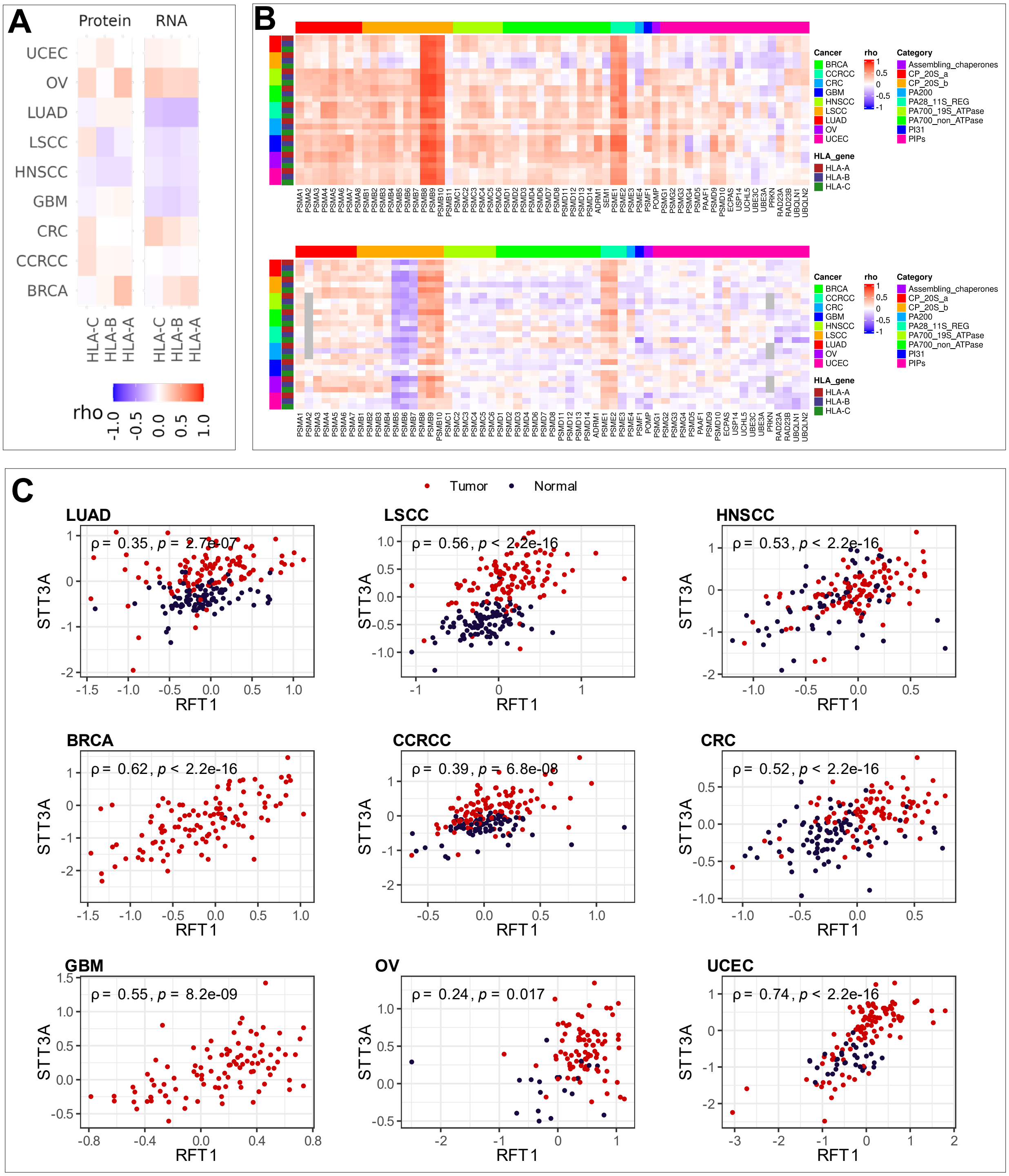
A
Protein
RNA
UCEC
OV
LUAD
LSCC
HNSCC
GBM
CRC
CCRCC
BRCA
HLA-C
HLA-B
HLA-A
rho
-1.0
-0.5
0.0
0.5
1.0
B
Cancer
BRCA
CCRCC
CRC
GBM
HNSCC
LSCC
LUAD
OV
UCEC
HLA_gene
HLA-A
HLA-B
HLA-C
rho
Category
Assembling_chaperones
CP_20S_a
CP_20S_b
PA200
PA28_11S_REG
PA700_19S_ATPase
PA700_non_ATPase
PI31
PIPs
C
Tumor
Normal
LUAD
ρ = 0.35, p = 2.7e-07
LSCC
ρ = 0.56, p < 2.2e-16
HNSCC
ρ = 0.53, p < 2.2e-16
BRCA
ρ = 0.62, p < 2.2e-16
CCRCC
ρ = 0.39, p = 6.8e-08
CRC
ρ = 0.52, p < 2.2e-16
GBM
ρ = 0.55, p = 8.2e-09
OV
ρ = 0.24, p = 0.017
UCEC
ρ = 0.74, p < 2.2e-16
STT3A
RFT1

Figure S8 Correlation between HLA-I and proteasome genes and mutation counts

(A) Correlation between HLA-I expression and tumor mutation counts

Tumor mutation counts were the numbers of somatic non-synonymous mutations identified with whole exome sequencing data, with MAF > 0.05. Correlation was calculated as Spearman's ρ between HLA-I gene RNA expression (upper) and protein expression (lower) with tumor mutation counts.

(B) Correlation between HLA-I and proteasome gene expression

Heatmap of Spearman's ρ between HLA-I RNA expression and proteasome gene RNA expression (upper) and HLA-I protein expression and proteasome gene protein expression(lower). Only tumor sample data were used in analysis.

(C) Correlation between RFT1 and STT3A protein expression in nine cancer types. Red: tumor samples; blue: normal samples. Correlations were calculated with Spearman's ρ and labeled on plots.
